# Genetic and molecular biology of breast cancer among Iranian patients

**DOI:** 10.1186/s12967-019-1968-2

**Published:** 2019-07-08

**Authors:** Meysam Moghbeli

**Affiliations:** Medical Genetics Research Center, Mashhad University of Medical Sciences, Mashhad, Iran

**Keywords:** Breast cancer, Diagnosis, Marker, Prognosis, Genetic, Risk factor

## Abstract

**Abstract:**

Background, Breast cancer (BC) is one of the leading causes of cancer related deaths in Iran. This high ratio of mortality had a rising trend during the recent years which is probably associated with late diagnosis.

**Main body:**

Therefore it is critical to define a unique panel of genetic markers for the early detection among our population. In present review we summarized all of the reported significant genetic markers among Iranian BC patients for the first time, which are categorized based on their cellular functions.

**Conclusions:**

This review paves the way of introducing a unique ethnic specific panel of diagnostic markers among Iranian BC patients. Indeed, this review can also clarify the genetic and molecular bases of BC progression among Iranians.

## Background

**Fig. 1:**
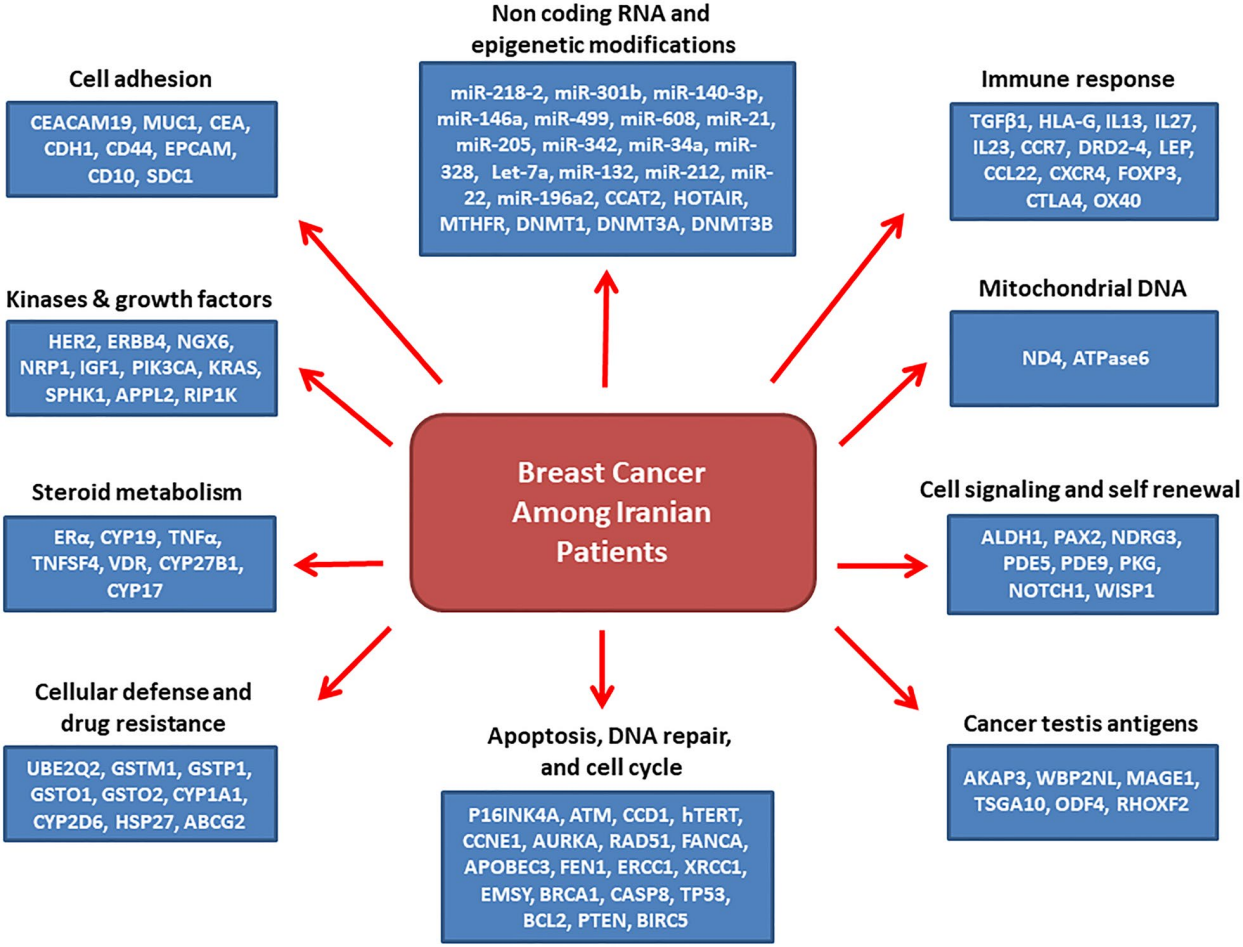
All the cellular processes which are involved in breast cancer progression among Iranian patients

Cancer is the second and third leading cause of deaths in developed and developing countries, respectively [1]. The China, India, and Japan with 59.7% of Asian breast cancer (BC) cases have the highest incidences in Asia [2]. BC is the most common cancer among Iranian females with mean age of between 46 and 49 years old [3]. Unfortunately, it has been shown that the BC mortality rate had a noticeable raising trend in 1990 to 2010 from 0.96 to 4.33 per 100,000 cases [4]. There is a variation about the BC incidence based on geographical districts in Iran in which the central provinces such as Tehran and Isfahan has the highest incidences [5]. Although, Iran is among the low incidence countries in the case of BC, the Iranian patients have lower ages and advanced stage tumors in comparison with another patients in western countries [6]. There are various BC risk factors which can be categorized as genetic and non-genetic. The non-genetic factors are age, life style, early menarche, late menopause, family history, weight, smoking, diet, socioeconomic condition, and air pollution. Age of diagnosis is about 40–50 years in Asian and African patients whereas it is about 60–70 years in Western countries [7, 8]. Ethnic is also important in age of diagnosis in which the black women have lower ages in comparison with the white women (58 vs. 62 years) [9]. Early Menarche/Late Menopause also increases the risk of breast cancer. Delayed pregnancy and decreased period of breastfeeding are also associated with elevated BC risk. Smoking before the menopause increases the risk of BC. There is also a direct correlation between alcohol consumption and BC risk. A regular physical activity and fruits and vegetables consumptions also decreases the BC risk. In the case of genetic risk factors, the BRCA1 and BRCA 2 mutations are among the main factors which are reported and involved in BC [10, 11]. Beside the BRCA family, the variety of genes are also reported during BC progression such as p53 and PTEN tumor suppressors [12, 13]. Members of DNA repair system and cell cycle regulators such as ATM, CHEK2, NBN, PALB2, and RAD50 are also correlated with risk of BC [14]. Late diagnosis in advanced stages of tumor is one of the main reason of high mortality among the BC patients. Therefore, finding new diagnostic markers will improve the early detection and better management of BC among patients. Regarding the differences between populations in the case of genetic factors, it is required to introduce a population specific panel of genetic markers for every population. Despite various studies about the role of different genes in BC progression among Iranians, there is not still any unique panel of markers for the early detection among Iranian patients. Therefore, for the first time in the present study we summarized the significant genetic markers which are reported until now among the Iranian BC patients (Table 1). We categorized these reported markers in different cellular processes based on their cellular functions (Fig. 1).

**Table 1:** All of the involved markers in BC susceptibility among the Iranian patients

Study (et al.)	Year	Gene	Type	Population	Results
Danesh [17]	2018	miR-218-2, miR-301b	Sporadic	288 Controls 266 Patients	Polymorphism was correlated with BC risk
Heydari [19]	2018	mir-140-3p	Sporadic	40 Controls 40 Patients	Over expression
Parchami Barjui [20]	2017	miR-502	Sporadic	231 Controls 240 Patients	Polymorphism was correlated with BC risk
Omrani [21]	2014	hsa-miR-499	Sporadic	203 Controls 236 Patients	Polymorphism was correlated with BC risk
Hashemi [23]	2016	mir-608	Sporadic	192 Controls 160 Patients	Polymorphism was correlated with BC risk
Savad [26]	2012	miR-342	Sporadic	77 N/T^a^	Over expression in ER/PR positive tumors
Mansoori [28]	2016	miR34a, let-7a	Sporadic	45 N/T	Under expression
Saberi [29]	2016	miR-328	Sporadic	28 N/T	Over expression
Damavandi [30]	2016	miR-132, miR-212, and miR-22	Sporadic	36 N/T	Correlation with grade and stage
Nejati-Azar [32]	2018	miR-196a	Sporadic	200 Controls 200 Patients	Polymorphism was correlated with BC risk
Sarrafzadeh [35]	2017	CCAT2	Familial/ Sporadic^b^	48 N/T	Correlation with stage an lymph node metastasis
Hassanzarei [37]	2017	HOTAIR	Sporadic	231 Controls 220 Patients	Polymorphism was correlated with BC risk
Miri [42]	2012	eRF3a/GSPT1	Familial/ Sporadic	250 Controls 250 Patients	Polymorphism was correlated with BC risk
Hosseini [45]	2011	MTHFR	Sporadic	306 Controls 294 Patients	Polymorphism was correlated with BC risk
Rezaei [46]	2017	MTHFR	Familial/ Sporadic	84 Patients	Polymorphism was correlated with BC risk
Jahangiri [48]	2018	DNMT1, DNMT3A, and DNMT3B	Sporadic	72 Patients	Correlation with grade
Eftekhar [49]	2014	DNMT3B	Sporadic	138 Controls 100 Patients	Polymorphism was correlated with BC risk
Hashemi [51]	2012	CASP8	Sporadic	203 Controls 236 Patients	Polymorphism was correlated with BC risk
Aghababazadeh [52]	2017	CASP8	Familial/ Sporadic	27 N/T	Under expression
Eskandari-Nasab [54]	2015	TP53	Sporadic	203 Controls 236 Patients	Polymorphism was correlated with BC risk
Payandeh [55]	2016	P53	Sporadic	231 Patients	Correlation with grade and lymph node metastasis
Rostamizadeh [58]	2013	BCL-2	Sporadic	40 Patients	Correlation with grade and survival
Golmohammadi [61]	2016	PTEN	Sporadic	100 Patients	Correlation with stage and survival
Sadeq [62]	2011	PTEN	Sporadic	20 Controls 57 Patients	Hypermethylation was correlated with stage and lymph node metastasis
Yari [63]	2016	PTEN	Familial/ Sporadic	50 Controls 103 Patients	Methylation
Ghaffari [64]	2016	BIRC5	Sporadic	40 Patients	Correlation with age
Vallian [70]	2009	p16INK4A	Sporadic	70 Controls 70 Patients	Methylation was correlated with stage
Salimi [72]	2012	ATM and cyclinD1	Sporadic	119 Patients	ATM under expression and cyclinD1 over expression
Mehdipour [73]	2011	ATM	Sporadic	248 Controls 129 Patients	Polymorphism was correlated with BC risk
Amininia [75]	2014	CCNE1	Sporadic	225 Controls 266 Patients	Polymorphism was correlated with BC risk
Hajikhan Mirzaei [78]	2012	DBC2	Sporadic	50 Patients	Methylation
Golmohammadi [79]	2017	AURKA	Sporadic	100 Controls 100 Patients	Polymorphism was correlated with BC risk
Mojgan [80]	2012	ERCC1	Sporadic	126 Controls 300 Patients	Polymorphism was correlated with BC risk
Jalali [83]	2016	XRCC1	Sporadic	200 Controls 100 Patients	Polymorphism was correlated with BC risk
Rezaei [86]	2016	FEN1	Sporadic	225 Controls 266 Patients	Mutation
Madjd [88]	2014	EMSY	Familial/ Sporadic	116 Patients	Correlation with tumor size and lymph node metastasis
Hallajian [91]	2017	ATM, RAD51, and BRCA1	Sporadic	50 Patients	Under expression
Darbeheshti [94]	2018	BRCA1	Sporadic	53 Patients	Correlation with grade and lymph node metastasis
Madjd [95]	2011	BRCA1	Familial/ Sporadic	156 Patients	Correlation with grade
Rezaei [96]	2015	APOBEC3	Sporadic	217 Controls 262 Patients	Mutation
Abbasi [98]	2017	FANCA	Familial/ Sporadic	295 Controls 304 Patients	Mutation
Hashemi [102]	2014	hTERT	Sporadic	225 Controls 266 Patients	Polymorphism was correlated with BC risk
Haghi [108]	2015	HLA-G	Sporadic	255 Controls 227 Patients	Polymorphism was correlated with BC risk
Mahmoodi [109]	2012	HLA-II	Sporadic	80 Controls 100 Patients	Polymorphism was correlated with BC risk
Ghaderi [110]	2001	HLADRB1	Sporadic	36 Controls 36 Patients	Polymorphism was correlated with BC risk
Faghih [113]	2009	IL13	Sporadic	195 Controls 305 Patients	Polymorphism was correlated with BC risk
Khodadadi [119]	2014	IL-27 and IL-23	Sporadic	50 Controls 50 Patients	Over expression
Vahedi [120]	2018	CCR7	Sporadic	70 N/T	Correlation with stage, grade, and lymph node metastasis
Jafarzadeh [123]	2015	CCL22	Sporadic	100 Controls 100 Patients	Correlation with stage
Dayer [124]	2018	CXCR4	Sporadic	36 N/T	Over expression in HER2 positive tumors
Jaberipour [127]	2010	FOXP3 and CTLA-4	Sporadic	40 Controls 55 Patients	Correlation with stage and grade
Hamidinia [128]	2013	FOXP3 and OX40	Sporadic	40 Controls 40 Patients	Correlation with stage
Rostami [134]	2015	Leptin	Sporadic	171 Controls 203 Patients	Polymorphism was correlated with BC risk
Mohammadzadeh [136]	2015	Leptin	Sporadic	100 Controls 100 Patients	Polymorphism was correlated with BC risk
Pornour [140]	2014	DRD2, DRD4	Sporadic	30 Controls 30 Patients	Over expression
Amani [141]	2014	TGFβ1	Sporadic	110 Controls 110 Patients	Polymorphism was correlated with BC risk
Parvizi [142]	2016	TGF-β	Sporadic	100 Controls 100 Patients	Polymorphism was correlated with BC risk
Tabatabaeian [143]	2013	HER-2	Familial/sporadic	15 Controls 80 Patients	Over expression
Panahi [144]	2013	HER-2	Familial	299 Patients	Correlation with ER/PR
Amirifard [145]	2016	HER-2	Familial/sporadic	130 Patients	Correlation with stage and age
Salimi [146]	2016	ERBB4	Familial/sporadic	192 Controls 172 Patients	Polymorphism was correlated with BC risk
Mansouri Bidkani [147]	2018	ERBB4	Familial/sporadic	148 Controls 172 Patients	Polymorphism was correlated with BC risk
Bagheri [148]	2016	ERBB4	Sporadic	146 Controls 146 Patients	Polymorphism was correlated with BC risk
Eskandari-Nasab [152]	2012	NGX6	Sporadic	203 Controls 236 Patients	Under expression
Seifi-Alan [154]	2018	NRP1	Sporadic	48 N/T	Correlation with stage, grade, and lymph node metastasis
Javadi [156]	2012	IGF-1	Familial/sporadic	224 Controls 215 Patients	Mutation
Azizi Tabesh [158]	2017	PIK3CA	Sporadic	80 Patients	Mutation
Sanaei [159]	2017	KRAS	Sporadic	204 Controls 244 Patients	Polymorphism was correlated with BC risk
Nazouri [160]	2017	SPHK1	Sporadic	120 Patients	Correlation with BMI
Heshmatpour [164]	2014	PIK3CA	Sporadic	200 Controls 200 Patients	Polymorphism was correlated with BC risk
Ghalaei [165]	2018	APPL2 and OCC1	Sporadic	30 N/T	APPL2 under expression and OCC1 over expression
Karami-Tehrani [166]	2016	RIP1K and RIP3K	Sporadic	20 Normal 45 Tumor	Correlation with grade
Nikseresht [168]	2010	UBE2Q2	Sporadic	21 N/T	Over expression
Hashemi [170]	2012	GSTM1, GSTT1, GSTP1	Sporadic	152 CONTROLS 134 PATIENTS	Polymorphism was correlated with BC risk
Sharif [171]	2017	GSTO1, GSTO2	Sporadic	150 Controls 153 Patients	Polymorphism was correlated with BC risk
Saadatian [173]	2014	CYP1A1	Sporadic	100 Controls 100 Patients	Polymorphism was correlated with BC risk
Saghafi [174]	2018	CYP2D6	Familial/ Sporadic	84 Patients	Polymorphism was correlated with BC risk
Yazdi [179]	2015	CYP2D6	Sporadic	101 Patients	Polymorphism was correlated with BC risk
Homaei-Shandiz [184]	2016	HSP27	Sporadic	65 Controls 97 Patients	High levels of serum anti Hsp27
Ghafouri [187]	2016	ABCG2	Sporadic	200 Controls 100 Patients	Polymorphism was correlated with BC risk
Estiar [189]	2017	CEACAM19	Sporadic	75 Patients	Over expression
Moazzezy [192]	2014	CA15-3 and CEA	Sporadic	30 Controls 30 Patients	Over expression
Mansouri [193]	2016	MUC1	Sporadic	50 Patients	Over expression
Zarei [196]	2017	CDH1	Sporadic	200 Controls 100 Patients	Polymorphism was correlated with BC risk
Esmaeili [198]	2018	CD44	Familial/ Sporadic	175 Controls 175 Patients	Polymorphism was correlated with BC risk
Sadeghi [202]	2017	EpCAM	Sporadic	122 Patients	Correlation with grade
Taghizadeh-Kermani [204]	2014	CD10	Sporadic	150 Patients	Correlation with grade, lymph node metastasis, and tumor size
Fard [207]	2018	CAV-1	Sporadic	203 Controls 203 Patients	Polymorphism was correlated with BC risk
Mobasheri [208]	2016	SYCP3	Sporadic	47 Patients	Over expression
Malek-Hosseini [209]	2017	SDC1	Familial/ Sporadic	30 Controls 61 Patients	Correlation with tumor size
Karami-Tehrani [213]	2012	PDE5 and PDE9	Sporadic	70 N/T	Correlation with stage, grade, and lymph node metastasis
Karami-Tehrani [215]	2012	PKG	Sporadic	70 N/T	Under expression
Paryan [218]	2016	NOTCH1	Sporadic	20 N/T	Correlation with tumor type
Taghavi [220]	2016	WISP1	Familial/ Sporadic	15 Controls 85 Patients	Correlation with age and tumor size
Madjd [224]	2012	ALDH1	Familial/ Sporadic	127 Patients	Correlation with BRCA1
Jahangiri [229]	2018	PAX2	Sporadic	72 Patients	Correlation with survival
Estiar [233]	2017	NDRG3	Sporadic	6 Normal 88 Tumor	Correlation with survival
Hosseini [234]	2014	ERα	Sporadic	10 Normal 19 Tumor	Over expression
Abbasi [235]	2013	ERα	Familial/ Sporadic	147 Controls 150 Patients	mutation
Farzaneh [236]	2016	CYP19	Sporadic	135 Controls 134 Patients	Polymorphism was correlated with BC risk
Kamali-Sarvestani [241]	2005	TNF-α	Sporadic	160 Patients	Polymorphism was correlated with BC risk
Vakil Monfared [245]	2018	TNFSF4	Sporadic	126 Controls 123 Patients	Polymorphism was correlated with BC risk
Colagar [250]	2015	VDR	Sporadic	127 Controls 134 Patients	Microsatellite
Zhalehjoo [255]	2017	CYP24A1 and CYP27B1	Sporadic	30 N/T	Correlation with stage and age
Shahabi [256]	2018	VDR	Familial/ Sporadic	214 Controls 203 Patients	Polymorphism was correlated with BC risk
Shahbazi [257]	2013	VDR	Sporadic	156 Controls 140 Patients	Polymorphism was correlated with BC risk
Ebrahimi [260]	2017	CYP17	Familial/ Sporadic	205 Patients	Polymorphism was correlated with BC risk
Esmaeili [263]	2015	AKAP3	Sporadic	162 N/T	Correlation with stage and tumor size
Nourashrafeddin [266]	2015	WBP2NL	Sporadic	30 Normal 50 Tumor	Over expression
Rastgoosalami [267]	2016	MAGE-1	Sporadic	113 Patients	Correlation with lymph node metastasis and tumor size
Dianatpour [268]	2012	TSGA10	Sporadic	50 Patients	Expression
Kazemi-Oula [272]	2015	ODF4 and RHOXF2	Familial/Sporadic	40 N/T	Over expression
Arezi [278]	2018	ND4	Sporadic	28 Normal 60 Tumor	Mutation
Ghaffarpour [282]	2014	ATPase6	Sporadic	49 Patients	Mutation

^a^Tumor tissues and normal margins

^b^Both of familial and sporadic patients among cases

## Main text

### Non coding RNAs and epigenetic modifications

MiRNAs as short non-coding RNAs can regulate gene expression post-transcriptionally through 3-UTR in the target mRNAs which are involved in various cellular functions including apoptosis and cell proliferation [15]. MiRNAs polymorphisms can change the function of miRNAs through modulation of their expression, processing, or binding sequences [16]. It has been observed that there was a significant correlation between rs11134527 variant in miR-218-2 and rs384262 variant in miR-301b and higher risk of BC among a sub population of Iranian patients [17]. SOX2 self renewal factor is one of the targets of miR-140 and miR-140/SOX2 interaction can be associated with cell survival in BC [18]. Another group also evaluated the serum levels of miR-140-3p expression in Iranian BC patients compared with healthy cases. They showed significant miR-140-3p over expression in BC in comparison with the normal cases. Moreover, there was a correlation between mir-140-3p expression and age, in which the premenopausal and ≤ 48 years old female with BC have shown mir-140-3p over expression. They introduced this marker as a diagnostic factor in premenopausal and in ≤ 48 years old females with BC [19]. BRCA1/BRCA2 are the targets of MiR-146a. It has been shown that the miR-146a is involved in BC. Moreover, there was a significant correlation between rs16917496 of miR-502 and higher risk of BC among a sub population of Iranian patients [20]. It has been shown that there was a correlation between hsa-miR-499 rs3746444 CC and C genotypes and high risk of BC among a subpopulation of Iranian subjects [21]. Mir-608 targets several factors such as INSR, IL-1A, GHR, and TP53 which are involved in tumor growth and apoptosis [22]. It has been observed that there was a significant correlation between rs4919510 polymorphism and BC, in which such polymorphism significantly decreased the BC susceptibility among a sub population of Iranian BC patients [23]. MiR-21 is involved in regulation of cell proliferation and apoptosis through targeting various factors such as PTEN and PDCD4 [24, 25]. Another study also showed that there was a high levels of miR-21 expression in aggressive breast tumors. Moreover, a significant under expression of miR-205 was observed in ER/PR/HER2 negative breast tumors compared with normal samples. The miR-342 under and over expressions were also observed in ER/PR negative and ER/PR positive BC tissues respectively in an Iranian population [26]. Although, MiR-34a and let-7 are as tumor suppressors are observed in normal cells, their under expression is also observed in tumors. MiR-34a is one of the targets of p53. MiR-34a also targets the BCL2 and caspase 3 [27]. It has been reported that the p53, miR34a, and let-7a were significantly down regulated in BC in comparison with normal samples, however, BCL2 was up regulated in a sub population of Iranian patients [28]. MiR-328 inhibits the BCRP/ABCG2 expression which is an ABC transporter protein involved in cellular defence and multi-drug resistance. It has been observed that the tumor tissues had higher levels of miR-328 expression in comparison with normal margins among a sub population of Iranian BC patients [29]. Significant miR-132/miR-212 downregulation and miR-22 upregulation were reported in BC samples compared with corresponding normal margins among Iranian cases. Moreover, highgrade samples had decreased expression of miR-132, miR-212, and miR-22, whereas the advanced stage (III) tumors had increased expression of these markers [30]. HOX10 transcription factor is one of the targets of mir196a2 [31]. It has been shown that the miR-196a expression in BC patients harboring C*/*C rs11614913 was higher than that in the cases with TT genotype. The rs11614913 polymorphism was introduced an early detection marker among a sub population of Iranian BC patients [32]. Long non-coding RNAs (lncRNA) are a class of transcripts longer than 200 nucleotides. Beside the aberration in coding RNAs, the deregulation of lncRNA is also related with tumor progression. They exerts their post-transcriptional regulatory functions through interaction with DNA, RNA, and protein molecules. CCAT2 is a lncRNA that exerts its oncogenic function through regulation of WNT/β-catenin pathway [33, 34]. It has been shown that there was lower levels of CCAT2 expression in tumor tissues compared with normal margins in a group of Iranian BC patients. Moreover, significant converse correlations were also observed between CCAT2 expression, stage, and lymph node involvement in which the early stage tumors without metastatic lymph nodes had higher levels of CCAT2 expression [35]. HOX transcript antisense intergenic RNA (HOTAIR) is also a lncRNA, which binds to LSD1 and PRC2 to promote HOXD repression through histone modification and chromatin remodeling [36]. It has been shown that the HOTAIR rs920778 and rs12826786 polymorphisms were significantly correlated with high risk of BC, and rs1899663 variant also showed a significant correlation with low risk of BC among a sub population of Iranian cases [37]. Therefore, HOTAIR have probably an important role in BC progression via polycomb complexes and can be introduced as efficient prognostic and diagnostic marker for the BC. Deregulation of translation machinery and ribosome biogenesis are common features of tumor cells in post transcriptional level and epigenetic. eRF1 and eRF3 are the main terminators in eukaryotic translation process, in which the RF3 functions as an enhancer of RF1 through GTPase activity [38]. Moreover, RF3 is involved in poly (A) tail stabilization via interaction with PABP [39]. RF3 has two isoforms including RF3a and RF3b that are encoded by eRF3a/GSPT1 and eRF3b/GSPT2 genes, respectively [40, 41]. The association between the GGC repeat polymorphism in exon 1 of eRF3a/GSPT1 and the potential genetic susceptibility for BC progression was evaluated among a sub population of Iranian subjects. It has been shown that the Iranian patients harboring the 12-GGC and 7-GGC alleles of eRF3a/GSPT1 have a high risk of BC. Moreover, the homozygote 7-GGC repeat can be correlated with the age of BC progression among a sub population of Iranian females [42]. The tumor progression is related with genetic and epigenetic changes. DNA methylation is one of the most important epigenetic mechanism during the neoplastic transformation. Role of DNA methylation in tumorigenesis is mainly related to the promoter hypomethylation and hypermethylation of oncogenes and tumor suppressor genes respectively. Methylenetetrahydrofolate reductase (MTHFR) regulates folate metabolism, DNA methylation, synthesis, and repair [43, 44]. There is probably a correlation between MTHFR 1298AA and 677CC genotype and higher risk of BC. Moreover, the frequency of MTHFR 677 CT, TT and 1298 CT, AA variants in BC patients were lower than that in the healthy controls in an Iranian population [45]. It has been also observed that the C677T TT variant of MTHFR was significantly correlated with elevated risk of familial BC in Iranian patients [46]. Tamoxifen has been used as a treatment option for hormone responsive breast tumors. However, about onethird of patients are resistant and face with relapse [47]. The expression of DNMT1, DNMT3A, and DNMT3B DNA methyltransferases in tamoxifen resistant patients were higher than that in the tamoxifen sensitive Iranian BC patients. There were significant associations between their protein expression and tumor grade, and also DNMT3B expression and lymph node metastasis in tamoxifen sensitive group. There was significant correlation between DNMT3A and DNMT3B expression and high histologic grade in tamoxifen resistant group. Generally they showed that the DNMTs overexpression has a negative association with survival in Iranian tamoxifen-treated BC cases [48]. Another group showed that, the DNMT3B 149 C → T polymorphism was significantly associated with reduced BC risk among patients in Southern Iran [49].

### Apoptosis, DNA repair, and cell cycle

Apoptosis is a critical process through the organ development and is a defense mechanism against deregulated cell proliferation and tumorigenesis. It includes several pathways such as extrinsic and intrinsic. Death receptors are the activators of extrinsic pathway which subsequently activates the caspase-8. Then, the caspase-8 initiates a cascade by cleaving caspase-3, caspase-7, or Bid [50]. A group evaluated the probable association between CASP8 6N del variant and BC susceptibility in Iranian patients. They showed that there was a significant higher frequency of del allele in the control group compared with BC cases, which highlights the anti tumori role of the CASP8 −652 6N del variant among Iranian BC cases [51]. Another group also assessed the levels of CASP8 expression in tumor in comparison with normal margins of Iranian BC patients, which showed decreased CASP8 expression in tumour tissue. Moreover, there was a significant correlation between CASP8 expression and HR status [52]. TP53 as a tumor suppressor regulates the cell cycle and apoptosis [53]. It was shown that the TP53 16-bp INS/DEL polymorphism can be associated with risk of BC among a sub population of Iranian patients [54]. There was also significant correlations between p53 expression, lymph node involvement, age, and tumor grade in Kurdish BC patients in Western Iran [55]. The mitochondrial pathway of apoptosis process is associated with BCL-2 which is also a prognostic factor in BC for its relation with defence mechanisms against the genotoxic damages [56, 57]. It has been observed that there was higher levels of BCL-2 mRNA expression in tumors compared with normal margins in a sub population of Iranian BC subjects. BCL-2 over expression was positively associated with tumor grade and ER/PR, and negatively with tumor size. There was also a significant correlation between high levels of BCL-2 mRNA expression and survival rates [58]. PTEN is a tumor suppressor via the stimulation of apoptosis in tumor cells and suppression of cell proliferation. Moreover, It is a negative regulator of intracellular PIP3 and AKT/PKB signaling pathway. PTEN regulates the p53 stability through AKT-MDM2 pathway or direct interaction [59]. Moreover, PTEN-p300 complex maintains P53 acetylation following the DNA damage [60]. A significant correlation between PTEN expression and stage has been reported among a group of Iranian BC patients, in which advanced stage tumors had lower levels of PTEN expression. Moreover, the tumors without PTEN expression had lower survival rates compared with tumors with PTEN expression, which can be introduced as a prognostic marker [61]. It has been observed that the majority of BC patients had PTEN promoter methylation which was not observed among the normal cases. Moreover, there was a correlation between PTEN hypermethylation and lymph node metastasis and advanced stages tumors among a sub population of Iranian patients [62]. Another study have reported there was association between PTEN promoter methylation and BC susceptibility among a sub population of Iranian patients [63]. BIRC5 is an anti apoptotic factor and cell cycle regulator which is only expressed at the embryonic stage. It has been reported that the BIRC5 is associated with early onset BC progression, in which there was a significant correlation between BIRC5 up regulation and Iranian patients who were younger than 40 years [64].

DNA repair and cell cycle regulation are important processes related to the cancer susceptibility and chemotherapeutic responses. Cell proliferation is regulated by signaling pathways to limit the proliferation in adequate ratio. The cell cycle is regulated by several factors such as cyclins, CDK, and CDKIs. G1 to S progression is related with cyclin D and CDK 4/6, which is repressed by INK4 family [65]. P16INK4A is belonged to the INK4 family [66], and its deregulation has been reported in different tumors [67]. The p16INK4A is responsible for the G0 and G1 cell cycle arrest through suppression of cyclin D–CDK4/6, and it also has a critical role as an inhibitor of pRb phosphorylation [68, 69]. It has been reported that there was a correlation between p16INK4A promoter methylation and primary BC, in which the hypermethylated cases were in primary tumors with stages of I, II and III. Therefore, they concluded that the p16INK4A promoter methylation can be introduced as an efficient marker of early detection among a sub population of Iranian sporadic BC patients [70]. Cyclin D1 is one of the main cell cycle regulators that binds with CDK4/6 to phosphorylate pRb through the cell cycle. The ataxia telangiectasia-mutated (ATM) is also involved in DNA damage recognition [71]. A study has evaluated the ATM and cyclin D1 expression in a sub population of Iranian BC patients. They showed about 21.6% of cyclin D1 under-expression which were mostly in HER-2–positive tumors. In the case of ATM, about 50% had ATM under-expression. The levels of ATM expression in normal margins were lower than normal controls which can be related to the epigenetic regulation of ATM expression. Therefore, they concluded that the ATM and cyclin D1 under and over expressions respectively can be associated with tumor progression among the Iranian BC patients [72]. It has been observed that there was a significant difference of D1853N ATM polymorphism between patients and healthy controls, introducing this polymorphism as a diagnostic marker among a sub population of Iranian cases [73]. Cyclins and CDKs activate the E2F transcription factor through inhibition of retinoblastoma protein. Cyclin E/CDK2 complex is associated with DNA replication and chromosomal stability [74]. A correlation between CCNE1 rs1406 C/A polymorphism and elevated risk of BC has been shown among an Iranian population in southeast of Iran [75]. DBC2 inhibits cell proliferation via CCND1 down-regulation [76], and is associated with ubiquitination, cell cycle, and apoptosis [77]. Methylation status of DBC2 promoter sequence was assessed in blood and BC tissue samples of an Iranian sub population, showed a correlation between aberrant DBC2 methylation and tumor progression in sporadic cases [78]. The AURKA is involved in cell cycle regulation, G2/M transition, centrosome division, and genomic instability. It has been observed that the BC cases had higher frequency of Ile/Ile variant at F31I locus of AURKA in comparison with the healthy cases. Moreover there was significant correlation between Ile/Ile F31I polymorphism and stage of BC among a sub population of Iranian patients [79]. ERCC1 as a member of nucleotide excision repair system is vital for the genome integrity. Association between ERCC1 C8092A and BC was assessed and showed a correlation between T/T of ERCC1 genotype and increased risk of BC among a sub population of Iranian patients [80]. XRCC1 is one of important factors in base excision repair to maintain the DNA against the carcinogens and oxidative species [81, 82]. It has been reported that the Arg194Trp XRCC1 polymorphism was more frequent among patients compared with normal cases. Moreover, this variant was also correlated with stage. They introduced the Trp194 allele of XRCC1 as a risk factor among Iranian Kurdish BC patients [83]. Flap endonuclease 1 (FEN1) tumor suppressor is a critical factor in DNA replication, repair, and genome stability [84]. It is involved in base–excision repair and formation of Okazaki fragments during DNA replication [85]. There was a correlation between FEN1 haplotypes and BC susceptibility among a sub population of Iranian patients in Southeastern Iran [86]. BRCA family mediates double stranded DNA break repairs and chromatin remodeling [87]. EMSY is an inhibitor of the BRCA2 transactivity. It has been shown that the majority of a group of Iranian BC patients had EMSY protein expression. The sporadic cases had higher levels of EMSY expression compared with familial cases. There was a direct correlation between EMSY expression, larger tumor size, higher relapse, and increased lymph node involvement [88]. Homologues Recombination (HR) is one of the main processes during the DSB repair. RAD51 is a key factor in HR regulation. DSB activates the ATM that subsequently phosphorylate and activate the BRCA1 which is associated with RAD51 [89, 90]. It has been shown that there were significant ATM, RAD51, and BRCA1 down regulations in BC tissues compared with normal margins in a sub population of Iranian subjects. ATM under expression was significantly correlated with tumor stage, vascular invasion, and malignant behavior. Down regulation of RAD51 was also significantly correlated with lymph nodes metastasis, age, and grade of tumor [91]. BRCA1 is a nuclear phosphoprotein involving in DSB repair, cell cycle regulation, and apoptosis [92, 93]. There was significant correlations between BRCA1 downregulation, high grade, and lymph node metastasis. Low levels of BRCA1 expression were observed in majority of triple-negative and luminal tumors in comparison with normal tissues among a sub population of Iranian cases [94]. The altered BRCA1 expression is more frequent in primary BC, and loss or decreased BRCA1 expression in high-grade were more frequent in comparison with differentiated tumors. Moreover, there was a significant inverse association between the levels of BRCA1 and CD44 expressions in Iranian BC patients [95]. APOBEC3 is a cytidine deaminase involved in RNA editing, cell cycle regulation, and repression of the retrovirus replication. It has been observed that the APOBEC3 deletion had a significant correlation with increased risk of BC among a sub population of Iranian cases [96]. The FANCA is a tumor suppressor related to DNA repair [97]. Duplication in FANCA promoter sequence can be associated with increased risk of BC among Iranian cases [98]. Telomeres are repeated sequences at the end of linear chromosomes to maintain them against the loss of genetic information through the cell proliferation [99]. Telomere shortening leads the cells up to the senescence and apoptosis, which is an intra cellular defence mechanism against genomic instability and tumorigenesis [100]. hTERT is the main subunit of telomerase complex to maintain the telomere stability [101]. The hTERT rs2736098 variant increased the risk of BC in a subpopulation of Iranian patients in southeast Iran [102].

### Immune response

The immune system is able to find tumor cells through tumor-specific antigens or other induced factors by cellular stress [103]. However the microenvironment changes by tumor cells can be resulted to tumor escape from immune response [104]. HLA system has important role in tumor antigen presentation for the immune system [105, 106]. HLA-G has immunosuppressive functions during the tumor progression [107]. A mutational analysis on a sub population of BC patients in northwestern-Iran showed a slightly higher allele frequency of HLA-G 14 bp deletion among patients. It seems that the high HLA-G expression induces tumor cells toward progressive stages. They concluded that the HLA-G 14 bp InDel polymorphism is probably a risk factor of BC progression among this population [108]. HLA-l and 2 present the antigens to the CD8 + T and CD4 + T cells, respectively. HLA-DRB1/DQB1 and HLA-DQA1 are belonged to the HLA-2 beta and alpha chains paralogues, respectively. It has been shown that the HLA-DQA1*0301 variant is correlated with elevated risk of early onset BC progression. Whereas, the HLADQA1*0505 and HLA-DQB1*0602 variants were associated with decreased risk of BC among young patients. They concluded that the HLA-II polymorphisms are risk factors of BC among a sub population of Iranian patients [109]. It has been observed that there was a significant association between HLADRB1* 12 allele and risk of BC among a sub population of Iranian patients [110]. Interleukin-13 is mainly secreted by activated TH2 lymphocytes and Natural Killer T cells [111, 112]. It has been shown that there was a correlation between CCA and ACA haplotypes of IL13 gene and risk of BC. Moreover, −1512C and −1055C alleles are involved in BC progression among a sub population of Iranian patients [113]. IL-27 is a heterodimeric cytokine comprising of EBI3 and P28 subunits that induces the CD4 + T cell proliferation through activation of STAT1 and 3 transcription factors [114, 115]. Moreover, IL-27 in association with TGF-β has a critical function in generation of IL-10-producing Tr1 cells [116]. IL-23 also regulates the memory CD4 + T cells proliferation through STAT3 activation [117, 118]. It has been reported that there was significantly higher levels of IL-27 and IL-23 expressions in BC peripheral bloods compared with normal cases, which can be introduced for the immunotherapy in Iranian BC patients [119]. CCR7 chemokine receptor is a G-protein coupled receptor involved in migration of immune cells to the lymphoid organs. It has been observed that the CCR7 expression was significantly associated with lymph node involvement, garde, and stage. Therefore, they introduced CCR7 as a prognostic marker among a sub population of Iranian BC patients [120]. CCL22 chemokine is produced by macrophages and DCs following the activation with microbial infection or CD40. The CCL22 production is regulated by Th1/2 cytokines such as IL-4, IL-5, and IFN-γ [121]. Therefore, CCL22 is associated with Th2- and Treg cells through inhibition of the immune responses against microbial infections and cancer cells [122]. It has been shown that the BC patients had significantly higher serum levels of CCL22 compared with healthy cases. There was also a direct correlation between the levels of CCL22 expression and stage of tumor. The frequencies of CC and C genotypes at rs223818 of CCL22 were significantly higher in BC patients in comparison with normal cases. Carriers of such genotypes also had higher serum levels of CCL22 compared with cases harboring GG or G variants, which highlighted the role of SNP rs223818 in BC risk via upregulation of the CCL22 [123]. CXCL12 is a chemokine that binds to its receptor (CXCR4) to activate MAPK1/MAPK3 through intracellular calcium ion which is involved in migration and adhesion. It has been shown that there was a correlation between HER2 and CXCR4 expression in a group of Iranian BC patients in which the HER2 positive tumors significantly had higher levels of CXCR4 expression in comparison with the HER2 negative tumors. They suggested the HER2 as probable inducer of CXCR4 among BC patients [124]. T-regulatory cells express cytotoxic T-lymphocyte antigen-4 (CTLA-4) and FoxP3 transcription factor [125, 126]. It has been shown that there was higher levels of FOXP3 and CTLA-4 mRNA expressions in blood samples of early stage and low grade BC patients compared with healthy subjects in an Iranian population. They highlighted the importance of regulatory T cells inhibitory mechanisms through the tumor progression which prepares opportunity for the tumor cells to hide from immune system [127]. A significant correlation has been also reported between FOXP3 and OX40 expressions and tumor stage in peripheral blood samples of Iranian BC patients [128]. Obesity is one of the main socio-epidemiological health problems globally and a negative BC prognostic factor [129]. The high ratio of adipose tissue increases the risk of tumor relapse in BC [130]. Negative effect of obesity on BC survival is associated with inflammatory cytokines such as leptin and IL-6 which are secreted by adipose tissue [131]. Leptin is an adipocytokine which is important in tumor progression through the activation of AKT signaling pathway [132]. Leptin is involved in cell proliferation, migration, and survival via regulation of various factors such as AP1, ERK2, and MAPK [133]. It has been reported that the leptin G-2548A (rs7799039) polymorphism was significantly correlated with risk of BC among a sub population of Iranian patients. They concluded that the females harboring AA genotype have an earlier menarche age and higher risk of breast cancer [134]. It has been also shown that the obese BC had higher serum leptin concentration compared with non-obese Iranian BC cases. Therefore, the inflammation has a key function in distant metastasis, since the adipose is the main source of leptin the weight loss through the physical activity can be an efficient option to decrease the negative role of leptin in BC progression [135]. LEP-2548G/A frequency was evaluated and showed the Iranian patients harboring LEP-2548G variant had a noticeable increased risk of BC [136]. Chronic stress is involved in tumorigenesis through secretion of neurotransmitters like during lymphocyte migration and angiogenesis. Dopamine is a neurotransmitter which has proliferative effects and induces the T cells for secretion of anti immune cytokines [137]. Dopamine receptors are from G-protein family which play important roles to induce or suppress intracellular cAMP [138]. The stress can alter dopamine receptor expression. Dopamine receptors (DRD1-DRD5) are categorised into D1 (stimulatory receptors) and D2 (inhibitory receptors) families [138, 139]. It has been reported that there was significantly higher levels of DRD2-DRD4 in a group of Iranian BC cases compared with healthy controls [140]. The transforming growth factor (TGF)-β is a pleiotropic cytokine expressed in several cells including peripheral blood mononuclear cells and platelets that plays critical roles in regulation of cell proliferation, apoptosis, and angiogenesis. The TGF-beta superfamily are involved in cell proliferation through activation of SMAD transcription factors. It has an anti cancer function in normal cells, whereas it promotes the tumor progression in advanced stage tumors. It has been observed that there was a significant difference in frequency of TGFβ1 “GTGCCGC” haplotype between BC and healthy controls among an Iranian population [141]. Moreover, it has been shown that there was significant correlations between TT and AA genotype of −509T/T and −800G/A TGF-β respectively and BC risk among a sub population of Iranian patients [142].

### Kinases and growth factors

The human epidermal growth factor 2 (HER-2) is a transmembrane tyrosine kinase receptor which is involved in cell proliferation. It was shown that fourteen out of 60 (23%) had HER-2 overexpression in a subpopulation of malignant BC patients in central Iran [143]. There was also an inverse correlation between HER-2 expression and ER/PR levels in a group of Iranian BC cases [144]. Another study on BC patients in Kermanshah province of Iran have shown a significant association between age, stage, and HER2-neu expression. Therefore, they introduced the HER2-neu as an efficient prognostic and diagnostic marker in that population [145]. ErbB4 is a tyrosine kinase receptor belonging to the EGFR subfamily. Frequency of rs11895168 variant was analyzed in a subpopulation of central Iranian BC patients, and observed that none of the normal cases harbored the rs11895168 C allele. They introduced a significant correlation between C allele and higher risk of breast cancer, which can be related to the miR-1276 binding changes [146]. Another study has shown that there was a significant correlation between rs13423759 allele C of ERBB4 and increased risk of BC which can be associated with role of this allele in strengthening the miR-548as-3p/ErbB4 interaction. The rs13423759 allele A is also significantly associated with decreased risk of breast cancer. Therefore, they highlighted the role of rs13423759 as an efficient diagnostic biomarker among a group of Iranian BC patients [147]. The rs1836724 polymorphism of ErbB4 was correlated with risk of BC, in which the T allele carriers had decreased expression of ErbB4 and higher susceptibility for BC among a group of Iranian patients [148]. NGX6 has an epidermal growth factor (EGF)-like domain which functions as a negative growth factor through apoptosis induction and cell proliferation arrest [149, 150]. NGX6 exerts its negative role on cell cycle via down regulation of cyclin D1, A, and E [151]. It has been shown that the levels of NGX6 mRNA expression in Iranian BC tumors were significantly lower than that in the normal samples [152]. Neuropilin-1 (NRP1) as a receptor for VEGF and PlGF in endothelial cells plays key roles in cell survival, angiogenesis, and EMT [153]. It has been shown that there was significant correlation between NRP1 expression and lymph node involvement in which the patients with metastatic lymph nodes had higher levels of NRP1 expression in comparison with the lymph node-negative cases. Moreover, the tumors with higher stages and grades had higher levels of NRP1 expressions. Therefore, they introduced NRP1 as a metastatic diagnostic marker among a sub population of Iranian BC patients [154]. Insulin-like growth factor 1 (IGF-1) is one of the activators of the AKT pathway, which is a cell proliferation inducer and cell death suppressor. There is a polymorphic cytosine–adenine (CA) repeat located in 1 kb upstream of the IGF-1 start site which is associated with cancer [155]. Length of this dinucleotide repeat was assessed in a group of Iranian BC patients. They showed that the patients harboring an allele longer than 19 and shorted than 20 have higher and lower risks of breast cancer, respectively. Therefore, there was a correlation between BC and length of the IGF-1 CA repeat which seems related to the ethnicity [156]. Phosphatidylinositol-3 kinase (PI3K) is a heterodimeric protein comprising of regulatory and catalytic subunits encoded by PIK3R1 and PIK3CA respectively, which is involved in apoptosis and cell proliferation [157]. Mutational analysis of PIK3CA catalytic subunit was done in a group of Iranian BC patients, and showed 45% of 80 cases had mutated PIK3CA that was higher than the recent reports. Majority of mutations were observed in three hotspots belonging to exon 9 and 20. There was a significant correlation between PIK3CA mutation and low grade tumors [158]. KRAS oncogene is a member of the small GTPase superfamily, which is also associated with PI3-kinase signaling pathway. It has been reported that the rs61764370 and rs712 polymorphisms in KRAS increased and decreases the BC risk in a sub population of Iranian patients [159]. SPHK1 catalyzes the phosphorylation of sphingosine to sphingosine-1-phosphate (S1P), which is a ligand of G protein-coupled receptors and regulates cell proliferation. SPHK1 exerts its inflammatory and anti apoptotic roles through several signaling pathways such as TNF-alpha and NF-kappa-B. It has been observed that there was a positive correlation between SPHK1 expression in ER/PR negative tumors compared with positive BC cases. Moreover, there was an association between BMI and SPHK1 expression among the Iranian ER negative BC patients [160]. The Class I PI3K family phosphorylates PIP2 to PIP3 which subsequently activates AKT as a regulator of cell proliferation, adhesion, and migration [161]. PIK3CA is the catalytic subunit of class I PI3Ks [162] that is deregulated in various cancers [163]. A highly polymorphic intronic GT dinucleotide repeat in PIK3CA had direct correlation with risk of BC in a group of Iranian patients, in which the harbors of short allele (17 repeats) and long allele (more than 16 repeats) had significantly higher and lower risk of BC progression, respectively [164]. OCC1 is involved in differentiation of adipocytes. APPL2 is one of the small GTPase RAB5A/Rab5 effectors, which are associated with endosomal membranes and can interact with the NuRD/MeCP1 complex which is required for cell proliferation. Levels of OCC1 and APPL2 expression were assessed in BC tissues, and showed significant under and over expressions of APPL2 and OCC1 in tumors compared with corresponding normal margins, respectively. Therefore, they suggested that the OCC-1D variant suppresses the APPL2 and probably regulate the cell cycle through PI3K/AKT signaling pathway [165]. RIP1K and RIP3K are belonged to RIPK family and involved in inflammation and cell death, in response to tissue damage. It has been observed that the benign and malignant tumors had increased levels of RIP1K compared with normal tissues. Whereas the malignant tumors had decreased levels of RIP3K expression compared with normal and benign samples. Moreover the RIP1K and RIP3K had decreased and increased expressions among old Iranian BC patients with malignant tumors, respectively. The tumors with higher grade and larger size had increased levels of RIP1K expression. Whereas the RIP3K had decreased expression in malignant tumors with grade III [166].

### Intracellular defense systems and drug resistance

Ubiquitin proteasome machinery is one of the main intracellular defence systems which is involved in degradation of aberrant and defective proteins. Moreover, it regulates the stability of cell cycle regulators. Ubiquitin–proteasome pathway involves several members such as Ubiquitin-activating (E1) and conjugating (E2) enzymes (E1) and ubiquitin ligases (E3). Since the lifetime regulation of cellular regulatory proteins including cell cycle and transcription factors is governed by the ubiquitin process, ubiquitine proteasome deregulation can be resulted in tumor progression [167]. UBE2Q2 expression was assessed in Iranian BC cases, and showed that the majority of tumor tissues had UBE2Q2 over expression compared with their corresponding normal margins. They introduced UBE2Q2 as a novel diagnostic marker among the Iranian BC patients [168]. Glutathione S-transferases (GSTs) as the cytoprotective factors detoxifie the carcinogens and chemotherapeutic drugs through the glutathione binding. Therefore, the human polymorphisms affects the individual ability to respond toward drugs and stresses. Although, the GSTM1 is expressed in a wide spectrum of tissues such as liver, stomach, and breast, GSTT1 enzyme is mainly observed in liver and erythrocytes [169]. The association between GSTM1, GSTT1 and GSTP1 genotypes and BC were assessed in a subpopulation of Iranian cases. It was reported that the GSTM1 null and GSTP1 Ile105Val genotypes were significantly correlated with the increased risk of BC. It seems that the cases with GSTM1 null, GSTT1 null, or GSTP1 Ile105Val Genotypes have low GST detoxification capacity, which can be resulted in higher accumulation of toxic compounds in such cases [170]. GSTO1 A140D and GSTO2 N142D polymorphisms were also associated with high risk of BC in a sub population of Iranian patients [171]. Cytochrome 450 (CYP450) is involved in oxidative catalysis of anticancer drugs and hormone synthesis. Therefore, assessment of CYP450 polymorphisms is required in anticancer therapy outcomes. CYP1A1 is involved in metabolism of xenobiotics and reactive epoxide production from aromatic compounds and steroid hormones [172]. It has been reported that the heterozygote carriers of 2455G (462Val) of CYP1A1 had significantly higher risk of BC in comparison with other genotypes. Moreover, heterozygote variant was more frequent in pre-menopausal patients, highlighting the role of this variation in early onset of BC among a group of Iranian patients in Eastern Azerbaijan [173]. Non functional CYP2D6*4 and 10 alleles were also frequent among BC patients in Northern Iran [174]. Tamoxifen is treatment option of ER-positive BC in premenopausal women [175, 176]. However, some patients do not response to the tamoxifen and die from tumor recurrence [177]. Tamoxifen is metabolized by cytochrome P450 2D6 (CYP2D6) in endoplasmic reticulum [178]. It has been shown that the HER2-neu positive Tamoxifen treated patients with CYP2D6 polymorphism had lower risk of tumor relapse in a sub population of Iranian BC patients [179]. Heat shock proteins (HSPs) are induced in response to anti-cancer drugs to maintain the cells in lethal conditions. Their cytoprotective function involve the correction of misfolded proteins and anti-apoptotic functions. Hsp27 regulates ability of cells to respond to the heat shock and oxidative stresses [180, 181]. Moreover, Hsp27 has an anti-apoptotic function through suppression of caspase-dependent apoptosis [182, 183]. It has been observed that there was a significant higher levels of serum anti- Hsp27 antibodies in BC compared with healthy Iranian cases [184]. The ATP-binding cassette (ABC) family regulates the drugs passage across cell membranes, which alters the kinetics of drugs. The ABCG2 is related with drug resistance in tumor cells and xenobiotic detoxification [185, 186]. It has been shown that there was a significant association between A allele of ABCG2 C421A variant and response to the Anthracyclines and Paclitaxol, and also increased BC risk in a group of Iranian BC patients [187].

### Cell adhesion, cytoskeletal factors, and ECM

Alterations in adhesion properties facilitate the tumor cell detachment from the primary tumor bulk and formation of secondary tumors in distant organs. The CEACAM subfamily are belonged to the CEA family which are involved in cell adhesion, differentiation, apoptosis, and angiogenesis [188]. Levels of CEACAM19 expression was evaluated among a group of Iranian BC patients, showing a higher levels of CEACAM19 mRNA expression in tumors compared with normal tissues [189]. The MUC1 is a mucin glycoproteins expressing on the apical surface of epithelial breast cells and is involved in cell adhesion [190]. CA15-3 serum assay determines the MUC1 protein. CEA is one of the members of immunoglobulin family and is associated with tumors [191]. Serum levels of CEA and CA15-3 were assessed in BC compared with healthy controls among Iranian cases and showed a significant increase of CA15-3 and CEA in BC in comparison with normal cases. There were significant correlations between CEA and grade of tumor, and also between CA15-3 and tumor size [192]. MUC1 overexpression was also correlated with BC progression and metastasis among Iranian cases [193]. CDH1 tumor suppressor is a cell–cell adhesion glycoprotein which is associated with cell differentiation and polarity [194, 195]. A significant correlation between CDH1 −160C/A polymorphism and BC susceptibility was reported among a group of Iranian Kurdish patients in which the A allele of CDH1 −160C/A variant was significantly associated with BC metastasis [196]. CD44 is involved normally in cell adhesion, migration, angiogenesis, and apoptosis [197]. The CD44 protein increases cell proliferation and migration in BC through an interaction with hyaluronan. It has been reported that there was a significant correlation between A > G intronic polymorphism and grade 3 of tumors in Iranian BC patients, which can be related to generation of a new SC35 binding site and modulation of splicing process. Low grade (1/2) tumors were also associated with AA genotypes [198]. Epithelial cell adhesion molecule (EpCAM) is a membrane glycoprotein associated with cell–cell adhesions and is involved in cell proliferation in different cancers [199]. EpCAM induces the Lef1 transcription factor through presenilin-2 to bind with promoter sequences of target genes such as c-myc and cyclin A/E genes [200]. C-myc protooncogene has important functions in various cellular processes such as cell cycle, angiogenesis, and apoptosis [201]. It has been observed that there was a significant correlation between EpCAM/c-myc over expression and grade III among a group of Iranian BC patients [202]. Matrix metalloproteinases are involved in ECM degradation to facilitate the cell migration and metastasis during the tumor progression. CD10 is a zinc-dependant metalloproteinase that is highly expressed in kidney and lung tissues and participates in ECM degradation [203]. Regulation of cell adhesion facilitates the tumor metastasis. Therefore, tumor cells regulate the cell adhesion through proteolytic enzymes or stimulation of fibroblasts to secrete such enzymes. It has been observed that there were inverse significant associations between stromal CD10 expression, larger tumor size, metastatic lymph nodes, and grade among Iranian BC patients [204]. Caveola are structural proteins in formation of caveola which are mainly expressed in endothelial, adipose, and smooth muscle cells [205]. CAV-1 is associated with tyrosine phosphorylation and observed in a wide spectrum of cells [206]. Role of several SNPs in CAV-1 was evaluated in BC progression among a group of Iranian patients, and showed that the cases harboring homozygous AA genotype in T29107A, G21985A, and G14713A polymorphisms have higher risk of BC progression compared with the normal cases. In contrast, the C521A homozygous variant had a protective role, and it had a significant correlation with BMI. Therefore, they introduced C521A, G14713A, G21985A, and T29107A as efficient diagnostic markers of BC progression among Iranian cases [207]. The SYCP3 is a structural component of the synaptonemal complex associated with recombination and meiotic chromosome segregation. There was a noticeable SYCP3 over expression in tumors compared with normal tissues. They introduced the SYCP3 as a CTA in BC which can be used as an early detection marker among Iranian BC patients [208]. Syndecan-1(SDC1) is an ECM receptor belonging to the heparan sulfate proteoglycan family. The extracellular domain binds to the heparan and chondroitin sulfate whereas the cytoplasmic domain is associated with cytoskeleton during the cell migration. It has been reported that there was elevated serum levels of SDC1 in BC in comparison with the healthy cases, which was also positively associated with size of BC tumors among Iranian patients [209].

### Cell signaling and self renewal

AMP-activated protein kinase (AMPK) is a cellular energy sensor, which is activated with elevated AMP/ATP ratio to induces the ATP generation. AMPK also acts as a tumor suppressor through cell cycle regulation. AMPK activates p53, which induces G1/S cell cycle arrest in response to the lack of glucose. Moreover, AMPK blocks CDKs via phosphorylation of p27 [210]. Levels of cAMP and cGMP as the second messengers are regulated via cyclic nucleotide phosphodiesterases (PDEs) [211]. PDE5 and PDE9 regulate the cGMP, which activates various downstream targets such as PKGs and ion channels [212]. Significant PDE5 and PDE9 over expressions has been observed in malignant tumors compared with benign tumors and normal margins. Moreover, there were significant correlations between phosphodiesterase expression and stage, grade and lymph node metastasis. Furthermore, there was a converse correlation between levels of expressions and age of patients. They concluded that the Iranian BC patients with higher levels of phosphodiesterases may have higher risks of malignant tumors [213]. Cyclic guanosine monophosphate (cGMP) participates in various signaling pathways through Protein Kinase G (PKG). PKGI and PKGII are serine/threonine kinases mediating the effects of cGMP [214]. It has been reported that the BC tissues had lower levels of PKG expression in comparison with the normal samples, highlighting the PKG as a tumor suppressor in a group of Iranian cases [215]. Notch is one of the main signaling pathways involved in cell proliferation, migration, and self renewal [216]. This pathway is a cell to cell contact process which is activated via the ligand binding with a family of trans membrane receptors (NOTCH1-4). Ligand binding releases the intracellular domain of Notch (ICN) into the cytoplasm which eventually enters to the nucleus where it regulated the expression of NOTCH target genes [217]. Levels of NOTCH1 mRNA expression as one of the NOTCH receptors was assessed in BC patients and showed a significant increase in invasive ductal types compared with other histopathological types. They introduced the NOTCH1 as a prognostic marker of IDC among Iranian patients [218]. WNT pathway is also another important process to maintain a normal balance between cell differentiation and proliferation. It is initiated through the cell surface receptors resulting in activation of cytoplasmic b-catenin which activates the LEF/TCF/PYGO2 transcriptional complex [219]. The WISP1 is belonged to the CTGF family and involved in WNT pathway. It also functions as an anti apoptotic factor through up regulation of BCL-X and inhibition of the P53 mediated apoptosis following the DNA damage via AKT activation. It has been reported that the metastatic BC tissues had significantly reduced levels of WISP1 mRNA expression in comparison with the normal subjects. Moreover, WISP1 expression was correlated with age and tumor size [220].

Cancer stem cells (CSC) are a small subpopulation of cells with self renewal and chemo-radiotherapeutic resistance abilities [221]. Aldehyde dehydrogenase 1 (ALDH1) has been suggested as a marker of mammary stem cells and prognostic marker [222, 223]. Epithelial and stromal expression of ALDH1 was analysed to assess the probable correlation between ALDH1 and clinicopathological features of breast tumors in a group of Iranian patients. They observed a significant converse correlation between ALDH1 and BRCA1 expressions. Moreover there was a correlation between ALDH1 +/BRCA1 low expression phenotype and high grade tumours [224]. BC cases are routinely undergone the different treatments such as surgery, radiotherapy, and chemotherapy. Hormone therapy is also one of the treatment options for the BC cases, in which the tamoxifen targets the positive estrogen receptor tumors through apoptosis induction and ER signaling inhibition [225, 226]. However, it has been observed that a noticeable ratio of tamoxifen treated cases have tumor relapse [227]. PAX2 is one of the members of PAX family of transcription factors which are involved in embryonic development of different organs [228]. PAX2 protein expression was assessed in tamoxifen treated BC patients, and showed that there was a correlation between PAX2 over expression and better survival. Moreover, the tamoxifen responsive cases had significantly higher levels of PAX2 expression compared with tamoxifen resistant patients [229]. MYC is an oncogene involving in various cellular processes, such as cell proliferation and apoptosis [230]. NDRG family is one of the targets of MYC which has four members including NDRG1-4 [231]. NDRG3 has high levels of expression in various tissues including prostate, testis, brain, heart, and kidney [232]. It has been suggested that the NDRG3 has a tumor suppressor role in BC patients, in which the triple negative and advanced stage BC tumors had the lowest levels of NDRG3 expression. Moreover, there was an inverse correlation between NDRG3 expression and survival rate, in which the higher levels of NDRG3 expression resulted in better survival rates among the Iranian BC patients [233].

### Vitamin D and steroids

Steroid hormones exert their regulatory role on transcription of target genes through binding with steroid receptors including estrogen receptor (ER) and progesterone receptor (PR). ERα has an important function in BC progression through cell growth and proliferation induction. It has been shown that there was an increased expression of ERα in the levels of mRNA and protein in a sub population of Iranian BC tissues in comparison with normal margins and it was associated with tumor size [234]. A mutational analysis has also shown that the presence of the ERα gene A908G mutation among a sub population of Iranian cases with invasive BC. Moreover, they observed a significant correlation between ER-α A908G mutation and familial BC [235]. P450 family is one the most important genes which are involved in steroid hormones metabolism. It has been observed that there was a significant association between rs10,046 polymorphism of CYP19 gene and BC among a group of Iranian patients [236]. TNF-α is involved in estrogen synthesis [237, 238] and down-regulation of ER [239]. Steroid hormone receptor is a prognostic factors to identify sensitive patients for hormone therapy, and also is associated with higher survival rates [240]. It has been shown that there was a significant correlation between TNFA −308 A/G polymorphism and levels of ER expression in a sub population of Iranian BC patients. Moreover, the cases harboring TNFA2 and TNFB2 alleles had lower risk of PR positive tumors in comparison with the TNFA1/A1 and TNFB1/B1 homozygous genotypes. They highlighted the correlation between immune and endocrine systems in BC progression, in which higher levels of TNF-α inhibits the PR expression [241]. TNFSF4 is also a glycoprotein belonged to the TNF family which is expressed on activated T cells and various antigen presenting cells such as dendritic cells and B cells [242–244]. It has been shown that the rs3850641 G allele is significantly correlated with an increased risk of BC among Iranian cases [245]. The inverse relation between 25-hydroxyvitamin D serum levels and BC can be related to the apoptotic and antiproliferative roles of vitamin D [246]. VDR is a nu-clear receptor for the steroid hormones participating in regulation target genes which are involved in cell proliferation [247, 248]. VDR/RXR complex binds to the promoter sequences of target genes to regulate their expression [249]. The length of VDR poly(A) microsatellite was assessed in a sub population of Iranian BC cases, and showed a significant correlation between increased BC risk and VDR poly(A) L variant [250]. This region is associated with mRNA stability, which highlights the key role of poly(A) microsatellite in regulation of VDR mRNA expression. CYP27B1 is involved in conversion of 25–hydroxyvitamin D3 to 1,25-dihydroxyvitamin D3 which finally regulates the transcription of target genes through VDR/RXR complex [251, 252]. This complex regulates the cell growth, apoptosis, and inflammation [253]. Finally the vitamin D pathway is terminated via CYP24A1 [254]. It has been reported that the levels of CYP27B1 mRNA expression in normal was higher than that in the tumor samples. In contrast, the tumors had significantly elevated levels of CYP24A1 expression compared with normal tissues. The CYP24A1 and CYP27B1 were significantly correlated with age and stage, respectively [255]. Another groups have also shown that there was a significant correlation between VDR BsmI polymorphism and elevated BC risk [256, 257]. CYP17 is a steroid monooxygenase which is involved in synthesis of glucocorticoids and sex steroids [258, 259]. It has been shown that the CYP17 MspA1 Polymorphism is probably age specific and is associated with increased risk of early onset BC among Iranian subjects [260].

### Cancer testis antigens

Cancer-testis antigens (CTA) are commonly expressed in testis and also with a lower ratio in ovarian germ cells [261]. Tumor cells also express antigens which are restricted to the germ cells. CTAs participate in EMT, self renewal, and tumor invasion [262]. AKAP as a group of CTAs has critical roles in sperm function which exerts their role through the protein kinase A (PKA). The levels of AKAP3 mRNA expression was assessed in tumor compared with normal margins of a sub population of Iranian BC cases, and showed a significant lower expressions in tumors compared with normal tissues. However, there was an inverse correlation between the levels of AKAP3 expressions and tumor sizes and stages, highlighting that as a probable inhibitor of proliferation [263]. WW-binding protein 2 (WBP2) as a CT antigen also regulates the expression of ERα/PR target genes through activation of ERα/PR expression in BC [264]. It has been demonstrated that the WBP2NL is probably associated with cell proliferation in embryonic stem cells and tumor cell lines [265]. WBP2NL over expression was also observed among the Iranian BC patients, highlighting that as a novel prognostic factor [266]. It has been reported that there was a significant correlation between MAGE-1 expression and lymph node involvement, and also between positive nuclear MAGE-1 expression and tumor size among Iranian BC patients [267]. Expressional analysis of TSGA10 CTA was performed in Iranian BC patients and showed its expression in 70% of patients while the only 12% of patients had immune response against TSGA10 [268]. ODF is involved in sperm tail maintenance and centrosome matrix [269] which facilitates the tumor cell proliferation. The RHOXF2 is associated with cell transformation [270] and cell to cell contacts [271]. The ODF4 and RHOXF2 cancer testis antigens had overexpression in tumors compared with normal marines in a sub population of Iranian BC patients [272].

### Mitochondrial DNA

Mitochondria as the powerhouse in the cells are involved in redox homeostasis, innate immunity, and apoptosis. Aberrant cellular energetics is one of the most critical risk factors during the tumor progression. Mitochondrial DNA mutations and enzyme defects are the main dysfunctions which cause the cellular energetic problems [273, 274]. The mitochondrial genome encodes various factors of respiratory chain and mitochondrial non coding RNAs [275]. ND4 as one of the respiratory chain components in mitochondria complex I (initiation of electron transport chain) is involved in various cancers [276]. Through the mitochondrial respiratory chain, reactive oxygen species (ROS) are generated due to the electron transfer, therefore it is really important to maintain the production and degradation of ROS, since there is not any protective factor in mitochondrial genome [277]. Mutational analysis of ND4 in a sub population of Iranian BC patients showed that there was a correlation between ND4 alteration and BC [278]. The mtDNA alterations are associated with the increased oxidative stresses and apoptotic resistance [279, 280]. The ATPase6 is belonged to the complex V genes, and contributed to the preservation of mtDNA [281]. The high frequency of ATPase6 mitochondrial mutations were observed in BC patients and highlighted the importance of mitochondrial gene variants in BC progression through modulation of metabolism, and can be introduced as molecular biomarkers among Iranian BC cases [282].

## Conclusions

For the first time in the current review we have summarized all of the recent significant genetic markers among Iranian BC patients. It seems that the non-coding RNAs, epigenetic modifications, and immune responses are the most common reported cell and molecular processes which are involved in tumor progression among Iranian BC patients. Moreover, we concluded that there were fifteen out of 117 reported genes which were assessed in higher numbers of BC patients and controls and they can be considered as the main high risk genes among Iranian BC patients (Table 2). Indeed, it is believed that the current review will be useful to pave the way of introducing a population based diagnostic panel markers for the early detection of BC among Iranians. Moreover, current review clarifies the molecular and genetic bases of BC progression in this population.

**Table 2:** The most reported and high risk genes among Iranian BC patients

Gene	Number of studies	Number of patients (total)	Number of controls (total)
CYP2D6	2	185	–
FOXP3	2	95	80
LEPTIN	2	303	271
TGFβ	2	210	210
HER2	3	509	15
ERBB4	3	490	486
CASP8	2	263	203
P53	2	467	203
PTEN	3	260	70
VDR	3	477	497
ATM	3	298	248
BRCA1	3	259	–
MTHFR	2	378	306
DNMT3	2	172	138
ERα	2	169	157

## Data Availability

The datasets used and/or analyzed during the current study are available from the corresponding author on reasonable request.

## Abbreviations

BC: breast cancer
GSTs: glutathione S-transferases
CYP450: cytochrome 450
HSPs: heat shock proteins
ABC: ATP-binding cassette
AMPK: AMP-activated protein kinase
PDEs: phosphodiesterases
cGMP: cyclic guanosine monophosphate
PKG: Protein Kinase G
ICN: intracellular domain of Notch
CTLA-4: cytotoxic T-lymphocyte antigen-4
TGF: transforming growth factor
HER-2: epidermal growth factor 2
EGF: epidermal growth factor
NRP1: neuropilin-1
IGF-1: insulin-like growth factor 1
CA: cytosine–adenine
PI3K: phosphatidylinositol-3 kinase
S1P: sphingosine-1-phosphate
ER: estrogen receptor
PR: progesterone receptor
lncRNA: long non-coding RNAs
HOTAIR: HOX transcript antisense intergenic RNA
ATM: ataxia telangiectasia–mutated
FEN1: flap endonuclease 1
HR: homologues recombination
CTA: cancer-testis antigens
PKA: protein kinase A
WBP2: WW-binding protein 2
ROS: reactive oxygen species
MTHFR: methylenetetrahydrofolate reductase
CSC: cancer stem cells
ALDH: aldehyde dehydrogenase 1
EpCAM: epithelial cell adhesion molecule
SDC1: Syndecan-1

## References

[1] Almasi Z, Rafiemanesh H, Salehiniya H (2015). Epidemiology characteristics and trends of incidence and morphology of stomach cancer in Iran. Asian Pac J Cancer Prev.

[2] Ghoncheh M, Momenimovahed Z, Salehiniya H (2016). Epidemiology, incidence and mortality of breast cancer in Asia. Asian Pac J Cancer Prev.

[3] Mousavi SM (2007). Breast cancer in Iran: an epidemiological review. Breast J.

[4] Sharifian A (2015). Burden of Breast Cancer in Iranian Women is Increasing. Asian Pac J Cancer Prev.

[5] Mahdavifar N (2016). Spatial analysis of breast cancer incidence in Iran. Asian Pac J Cancer Prev.

[6] Afsharfard A (2013). Trends in epidemiology, clinical and histopathological characteristics of breast cancer in Iran: results of a 17 year study. Asian Pac J Cancer Prev.

[7] Abdulrahman GO, Rahman GA (2012). Epidemiology of breast cancer in Europe and Africa. J Cancer Epidemiol.

[8] Leong SP (2010). Is breast cancer the same disease in Asian and Western countries?. World J Surg.

[9] Iqbal J (2015). Differences in breast cancer stage at diagnosis and cancer-specific survival by race and ethnicity in the United States. JAMA.

[10] Welcsh PL, King MC (2001). BRCA1 and BRCA2 and the genetics of breast and ovarian cancer. Hum Mol Genet.

[11] Xu X (1999). Conditional mutation of Brca1 in mammary epithelial cells results in blunted ductal morphogenesis and tumour formation. Nat Genet.

[12] Sidransky D (1992). Inherited p53 gene mutations in breast cancer. Cancer Res.

[13] Tan MH (2012). Lifetime cancer risks in individuals with germline PTEN mutations. Clin Cancer Res.

[14] Walsh T, King MC (2007). Ten genes for inherited breast cancer. Cancer Cell.

[15] Bartel DP (2004). MicroRNAs: genomics, biogenesis, mechanism, and function. Cell.

[16] Ryan BM, Robles AI, Harris CC (2010). Genetic variation in microRNA networks: the implications for cancer research. Nat Rev Cancer.

[17] Danesh H (2018). Association study of miR-100, miR-124-1, miR-218-2, miR-301b, miR-605, and miR-4293 polymorphisms and the risk of breast cancer in a sample of Iranian population. Gene.

[18] Zhang Y (2012). Estrogen receptor alpha signaling regulates breast tumor-initiating cells by down-regulating miR-140 which targets the transcription factor SOX2. J Biol Chem.

[19] Heydari N (2018). Overexpression of serum MicroRNA-140-3p in premenopausal women with newly diagnosed breast cancer. Gene.

[20] Parchami Barjui S (2017). Study of correlation between genetic variants in three microRNA genes (hsa-miR-146a, hsa-miR-502 binding site, hsa-miR-27a) and breast cancer risk. Curr Res Transl Med.

[21] Omrani M (2014). Hsa-mir-499 rs3746444 gene polymorphism is associated with susceptibility to breast cancer in an Iranian population. Biomark Med.

[22] Landi D (2008). A catalog of polymorphisms falling in microRNA-binding regions of cancer genes. DNA Cell Biol.

[23] Hashemi M (2016). miR-608 rs4919510 C > G polymorphism decreased the risk of breast cancer in an Iranian subpopulation. Exp Oncol.

[24] Papagiannakopoulos T, Shapiro A, Kosik KS (2008). MicroRNA-21 targets a network of key tumor-suppressive pathways in glioblastoma cells. Cancer Res.

[25] Qi L (2009). Expression of miR-21 and its targets (PTEN, PDCD4, TM1) in flat epithelial atypia of the breast in relation to ductal carcinoma in situ and invasive carcinoma. BMC Cancer.

[26] Savad S (2012). Expression analysis of MiR-21, MiR-205, and MiR-342 in breast cancer in Iran. Asian Pac J Cancer Prev.

[27] Zenz T (2009). miR-34a as part of the resistance network in chronic lymphocytic leukemia. Blood.

[28] Mansoori B (2016). Micro RNA 34a and Let-7a expression in human breast cancers is associated with apoptotic expression genes. Asian Pac J Cancer Prev.

[29] Saberi A (2016). MiR-328 may be considered as an oncogene in human invasive breast carcinoma. Iran Red Crescent Med J.

[30] Damavandi Z (2016). Aberrant expression of breast development-related microRNAs, miR-22, miR-132, and miR-212, breast tumor tissues. J Breast Cancer.

[31] Ma XP (2013). Association between microRNA polymorphisms and cancer risk based on the findings of 66 case-control studies. PLoS ONE.

[32] Nejati-Azar A, Alivand MR (2018). miRNA 196a2(rs11614913) & 146a(rs2910164) polymorphisms & breast cancer risk for women in an Iranian population. Per Med.

[33] Cai Y, He J, Zhang D (2015). Long noncoding RNA CCAT2 promotes breast tumor growth by regulating the Wnt signaling pathway. Onco Targets Ther.

[34] Ling H (2013). CCAT2, a novel noncoding RNA mapping to 8q24, underlies metastatic progression and chromosomal instability in colon cancer. Genome Res.

[35] Sarrafzadeh S (2017). Expression study and clinical correlations of MYC and CCAT2 in breast cancer patients. Iran Biomed J.

[36] Gupta RA (2010). Long non-coding RNA HOTAIR reprograms chromatin state to promote cancer metastasis. Nature.

[37] Hassanzarei S (2017). Genetic polymorphisms of HOTAIR gene are associated with the risk of breast cancer in a sample of southeast Iranian population. Tumour Biol.

[38] Zhouravleva G (1995). Termination of translation in eukaryotes is governed by two interacting polypeptide chain release factors, eRF1 and eRF3. EMBO J.

[39] Hoshino S (1999). Novel function of the eukaryotic polypeptide-chain releasing factor 3 (eRF3/GSPT) in the mRNA degradation pathway. Biochemistry (Mosc).

[40] Hansen LL, Jakobsen CG, Justesen J (1999). Assignment of the human peptide chain release factor 3 (GSPT2) to Xp11.23– > p11.21 and of the distal marker DXS1039 by radiation hybrid mapping. Cytogenet Cell Genet..

[41] Ozawa K (1992). Mapping of the human GSPT1 gene, a human homolog of the yeast GST1 gene, to chromosomal band 16p131. Somat Cell Mol Genet.

[42] Miri M (2012). GGCn polymorphism of eRF3a/GSPT1 gene and breast cancer susceptibility. Med Oncol.

[43] Das PM, Singal R (2004). DNA methylation and cancer. J Clin Oncol.

[44] Ueland PM (2001). Biological and clinical implications of the MTHFR C677T polymorphism. Trends Pharmacol Sci.

[45] Hosseini M, Houshmand M, Ebrahimi A (2011). MTHFR polymorphisms and breast cancer risk. Arch Med Sci.

[46] Rezaei H, Rassi H, Mansur FN (2017). Investigation of Methylenetetrahydrofolate reductase C677T polymorphism and human papilloma virus genotypes in Iranian breast cancer. Monoclon Antib Immunodiagn Immunother.

[47] Johansson HJ (2013). Retinoic acid receptor alpha is associated with tamoxifen resistance in breast cancer. Nat Commun.

[48] Jahangiri R (2018). Expression and clinicopathological significance of DNA methyltransferase 1, 3A and 3B in tamoxifen-treated breast cancer patients. Gene.

[49] Eftekhar E (2014). The study of DNA methyltransferase-3B promoter variant genotype among iranian sporadic breast cancer patients. Iran J Med Sci.

[50] Ghavami S (2009). Apoptosis and cancer: mutations within caspase genes. J Med Genet.

[51] Hashemi M (2012). Bi-directional PCR allele-specific amplification (bi-PASA) for detection of caspase-8 -652 6 N ins/del promoter polymorphism (rs3834129) in breast cancer. Gene.

[52] Aghababazadeh M (2017). Downregulation of Caspase 8 in a group of Iranian breast cancer patients—a pilot study. J Egypt Natl Canc Inst.

[53] Parant JM, Lozano G (2003). Disrupting TP53 in mouse models of human cancers. Hum Mutat.

[54] Eskandari-Nasab E (2015). Effect of TP53 16-bp and beta-TrCP 9-bp INS/DEL polymorphisms in relation to risk of breast cancer. Gene.

[55] Payandeh M (2016). Expression of p53 breast cancer in kurdish women in the West of Iran: a reverse correlation with lymph node metastasis. Asian Pac J Cancer Prev.

[56] Chipuk JE (2010). *The BCL*-*2 family reunion*. Mol Cell.

[57] Martinou JC, Youle RJ (2011). Mitochondria in apoptosis: Bcl-2 family members and mitochondrial dynamics. Dev Cell.

[58] Rostamizadeh L (2013). Bcl-2 gene expression in human breast cancers in iran. Asian Pac J Cancer Prev.

[59] Freeman DJ (2003). PTEN tumor suppressor regulates p53 protein levels and activity through phosphatase-dependent and -independent mechanisms. Cancer Cell.

[60] Li AG (2006). Mechanistic insights into maintenance of high p53 acetylation by PTEN. Mol Cell.

[61] Golmohammadi R (2016). Prognostic role of PTEN gene expression in breast cancer patients from north-east Iran. Asian Pac J Cancer Prev.

[62] Sadeq V, Isar N, Manoochehr T (2011). Association of sporadic breast cancer with PTEN/MMAC1/TEP1 promoter hypermethylation. Med Oncol.

[63] Yari K, Payandeh M, Rahimi Z (2016). Association of the hypermethylation status of PTEN tumor suppressor gene with the risk of breast cancer among Kurdish population from Western Iran. Tumour Biol.

[64] Ghaffari K (2016). BIRC5 genomic copy number variation in early-onset breast cancer. Iran Biomed J.

[65] Rocco JW, Sidransky D (2001). p16(MTS-1/CDKN2/INK4a) in cancer progression. Exp Cell Res.

[66] Serrano M, Hannon GJ, Beach D (1993). A new regulatory motif in cell-cycle control causing specific inhibition of cyclin D/CDK4. Nature.

[67] Nielsen NH (2001). Methylation of the p16(Ink4a) tumor suppressor gene 5′-CpG island in breast cancer. Cancer Lett.

[68] Kannan K (2003). Components of the Rb pathway are critical targets of UV mutagenesis in a murine melanoma model. Proc Natl Acad Sci USA.

[69] Malumbres M (2000). Cellular response to oncogenic ras involves induction of the Cdk4 and Cdk6 inhibitor p15(INK4b). Mol Cell Biol.

[70] Vallian S (2009). Methylation status of p16 INK4A tumor suppressor gene in Iranian patients with sporadic breast cancer. J Cancer Res Clin Oncol.

[71] Khanna KK (2000). Cancer risk and the ATM gene: a continuing debate. J Natl Cancer Inst.

[72] Salimi M, Mozdarani H, Majidzadeh K (2012). Expression pattern of ATM and cyclin D1 in ductal carcinoma, normal adjacent and normal breast tissues of Iranian breast cancer patients. Med Oncol.

[73] Mehdipour P (2011). Importance of ATM gene as a susceptible trait: predisposition role of D1853 N polymorphism in breast cancer. Med Oncol.

[74] Spruck CH, Won KA, Reed SI (1999). Deregulated cyclin E induces chromosome instability. Nature.

[75] Amininia S (2014). Association between CCNE1 polymorphisms and the risk of breast cancer in a sample of southeast Iranian population. Med Oncol.

[76] Yoshihara T, Collado D, Hamaguchi M (2007). Cyclin D1 down-regulation is essential for DBC2′s tumor suppressor function. Biochem Biophys Res Commun.

[77] Siripurapu V (2005). DBC2 significantly influences cell-cycle, apoptosis, cytoskeleton and membrane-trafficking pathways. J Mol Biol.

[78] Hajikhan Mirzaei M (2012). Evaluation of Methylation Status in the 5′UTR Promoter Region of the DBC2 Gene as a Biomarker in Sporadic Breast Cancer. Cell J.

[79] Golmohammadi R (2017). A single nucleotide polymorphism in codon F31I and V57I of the AURKA gene in invasive ductal breast carcinoma in Middle East. Medicine (Baltimore).

[80] Mojgan H, Massoud H, Ahmad E (2012). ERCC1 intron 1 was associated with breast cancer risk. Arch Med Sci.

[81] Dai L (2009). XRCC1 gene polymorphisms and esophageal squamous cell carcinoma risk in Chinese population: a meta-analysis of case–control studies. Int J Cancer.

[82] Yu H (2011). Interaction between XRCC1 polymorphisms and intake of long-term stored rice in the risk of esophageal squamous cell carcinoma: a case-control study. Biomed Environ Sci.

[83] Jalali C (2016). Association of XRCC1 Trp194 allele with risk of breast cancer, and Ki67 protein status in breast tumor tissues. Saudi Med J.

[84] Liu L (2012). Functional FEN1 genetic variants contribute to risk of hepatocellular carcinoma, esophageal cancer, gastric cancer and colorectal cancer. Carcinogenesis.

[85] Shen B (2005). Multiple but dissectible functions of FEN-1 nucleases in nucleic acid processing, genome stability and diseases. BioEssays.

[86] Rezaei M (2016). FEN1 -69G > A and +4150G > T polymorphisms and breast cancer risk. Biomed Rep.

[87] Venkitaraman AR (2002). Cancer susceptibility and the functions of BRCA1 and BRCA2. Cell.

[88] Madjd Z (2014). Expression of EMSY, a novel BRCA2-link protein, is associated with lymph node metastasis and increased tumor size in breast carcinomas. Asian Pac J Cancer Prev.

[89] Gatei M (2000). Role for ATM in DNA damage-induced phosphorylation of BRCA1. Cancer Res.

[90] Moynahan ME (1999). Brca1 controls homology-directed DNA repair. Mol Cell.

[91] Hallajian Z, Mahjoubi F, Nafissi N (2017). Simultaneous ATM/BRCA1/RAD51 expression variations associated with prognostic factors in Iranian sporadic breast cancer patients. Breast Cancer.

[92] Deng CX (2006). BRCA1: cell cycle checkpoint, genetic instability, DNA damage response and cancer evolution. Nucleic Acids Res.

[93] Paterson JW (1998). BRCA1: a review of structure and putative functions. Dis Markers.

[94] Darbeheshti F (2018). Comparison of BRCA1 expression between triple-negative and luminal breast tumors. Iran Biomed J.

[95] Madjd Z (2011). *BRCA1 protein expression level and CD44(*+*)phenotype in breast cancer patients*. Cell J.

[96] Rezaei M (2015). APOBEC3 deletion is associated with breast cancer risk in a sample of southeast Iranian population. Int J Mol Cell Med.

[97] Hussain S (2003). Direct interaction of the Fanconi anaemia protein FANCG with BRCA2/FANCD1. Hum Mol Genet.

[98] Abbasi S, Rasouli M (2017). A rare FANCA gene variation as a breast cancer susceptibility allele in an Iranian population. Mol Med Rep.

[99] Basu N (2013). Telomeres and telomere dynamics: relevance to cancers of the GI tract. Expert Rev Gastroenterol Hepatol.

[100] Vodenicharov MD, Wellinger RJ (2007). The cell division cycle puts up with unprotected telomeres: cell cycle regulated telomere uncapping as a means to achieve telomere homeostasis. Cell Cycle.

[101] Gomez DE (2012). Telomere structure and telomerase in health and disease (review). Int J Oncol.

[102] Hashemi M (2014). Association between hTERT polymorphisms and the risk of breast cancer in a sample of Southeast Iranian population. BMC Res Notes.

[103] Burnet M (1957). Cancer; a biological approach. I. The processes of control. Br Med J.

[104] Elliott RL (2011). Human leukocyte antigen G expression in breast cancer: role in immunosuppression. Cancer Biother Radiopharm.

[105] Pistillo MP (2000). Biochemical analysis of HLA class I subunits expression in breast cancer tissues. Hum Immunol.

[106] Salih HR, Nussler V (2001). Commentary: immune escape versus tumor tolerance: how do tumors evade immune surveillance?. Eur J Med Res.

[107] Tripathi P, Agrawal S (2006). Non-classical HLA-G antigen and its role in the cancer progression. Cancer Invest.

[108] Haghi M (2015). 14-bp insertion/deletion polymorphism of the HLA-G gene in breast cancer among women from north western Iran. Asian Pac J Cancer Prev.

[109] Mahmoodi M (2012). HLA-DRB1,-DQA1 and -DQB1 allele and haplotype frequencies in female patients with early onset breast cancer. Pathol Oncol Res.

[110] Ghaderi A (2001). HLA-DBR 1 alleles and the susceptibility of Iranian patients with breast cancer. Pathol Oncol Res.

[111] Hershey GK (2003). IL-13 receptors and signaling pathways: an evolving web. J Allergy Clin Immunol.

[112] Wynn TA (2003). IL-13 effector functions. Annu Rev Immunol.

[113] Faghih Z (2009). Interleukin13 haplotypes and susceptibility of Iranian women to breast cancer. Mol Biol Rep.

[114] Owaki T (2005). A role for IL-27 in early regulation of Th1 differentiation. J Immunol.

[115] Pflanz S (2002). IL-27, a heterodimeric cytokine composed of EBI3 and p28 protein, induces proliferation of naive CD4 + T cells. Immunity.

[116] Oppmann B (2000). Novel p19 protein engages IL-12p40 to form a cytokine, IL-23, with biological activities similar as well as distinct from IL-12. Immunity.

[117] Aggarwal S (2003). Interleukin-23 promotes a distinct CD4 T cell activation state characterized by the production of interleukin-17. J Biol Chem.

[118] Zheng Y (2007). Interleukin-22, a T(H)17 cytokine, mediates IL-23-induced dermal inflammation and acanthosis. Nature.

[119] Khodadadi A (2014). IL-23/IL-27 Ratio in Peripheral Blood of Patients with Breast Cancer. Iran J Med Sci.

[120] Vahedi L (2018). Investigation of CCR120 marker expression using immunohistochemical method and its association with clinicopathologic properties in patients with breast cancer. Int J Hematol Oncol Stem Cell Res.

[121] Yamashita U, Kuroda E (2002). Regulation of macrophage-derived chemokine (MDC, CCL22) production. Crit Rev Immunol.

[122] Nishikawa H, Sakaguchi S (2010). Regulatory T cells in tumor immunity. Int J Cancer.

[123] Jafarzadeh A (2015). Higher circulating levels of chemokine CCL22 in patients with breast cancer: evaluation of the influences of tumor stage and chemokine gene polymorphism. Tumour Biol.

[124] Dayer R (2018). Upregulation of CXC chemokine receptor 4-CXC chemokine ligand 12 axis ininvasive breast carcinoma: a potent biomarker predicting lymph node metastasis. J Cancer Res Ther.

[125] Fontenot JD (2005). Regulatory T cell lineage specification by the forkhead transcription factor foxp3. Immunity.

[126] Hori S, Nomura T, Sakaguchi S (2003). Control of regulatory T cell development by the transcription factor Foxp3. Science.

[127] Jaberipour M (2010). Increased CTLA-4 and FOXP3 transcripts in peripheral blood mononuclear cells of patients with breast cancer. Pathol Oncol Res.

[128] Hamidinia M (2013). Concomitant Increase of OX40 and FOXP3 transcripts in peripheral blood of patients with breast cancer. Iran J Immunol.

[129] Protani M, Coory M, Martin JH (2010). Effect of obesity on survival of women with breast cancer: systematic review and meta-analysis. Breast Cancer Res Treat.

[130] Iyengar NM, Hudis CA, Dannenberg AJ (2013). Obesity and inflammation: new insights into breast cancer development and progression. Am Soc Clin Oncol Educ Book.

[131] Pierce BL (2009). Elevated biomarkers of inflammation are associated with reduced survival among breast cancer patients. J Clin Oncol.

[132] Vona-Davis L, Rose DP (2007). Adipokines as endocrine, paracrine, and autocrine factors in breast cancer risk and progression. Endocr Relat Cancer.

[133] Guo S (2012). Oncogenic role and therapeutic target of leptin signaling in breast cancer and cancer stem cells. Biochim Biophys Acta.

[134] Rostami S, Kohan L, Mohammadianpanah M (2015). The LEP G-2548A gene polymorphism is associated with age at menarche and breast cancer susceptibility. Gene.

[135] Babaei Z (2015). Relationship of obesity with serum concentrations of leptin, CRP and IL-6 in breast cancer survivors. J Egypt Natl Canc Inst.

[136] Mohammadzadeh G (2015). The relationship between -2548 G/A Leptin gene polymorphism and risk of breast cancer and serum leptin levels in Ahvazian women. Iran J Cancer Prev.

[137] Basu B (2010). D1 and D2 dopamine receptor-mediated inhibition of activated normal T cell proliferation is lost in jurkat T leukemic cells. J Biol Chem.

[138] Beaulieu JM, Gainetdinov RR (2011). The physiology, signaling, and pharmacology of dopamine receptors. Pharmacol Rev.

[139] Kirillova GP (2008). Dopamine receptors in human lymphocytes: radioligand binding and quantitative RT-PCR assays. J Neurosci Methods.

[140] Pornour M (2014). Dopamine receptor gene (DRD1-DRD5) expression changes as stress factors associated with breast cancer. Asian Pac J Cancer Prev.

[141] Amani D (2014). Transforming growth factor beta1 (TGFbeta1) polymorphisms and breast cancer risk. Tumour Biol.

[142] Parvizi S (2016). Effects of two common promoter polymorphisms of transforming growth factor-beta1 on breast cancer risks in Ahvaz, west south of Iran. Iran J Cancer Prev.

[143] Tabatabaeian H, Hojati Z (2013). Assessment of HER-2 gene overexpression in Isfahan province breast cancer patients using Real Time RT-PCR and immunohistochemistry. Gene.

[144] Panahi M (2013). Expressional correlation of human epidermal growth factor receptor 2, estrogen/progesterone receptor and protein 53 in breast cancer. Asian Pac J Cancer Prev.

[145] Amirifard N (2016). Relationship between HER2 proto-oncogene status and prognostic factors of breast cancer in the west of Iran. Asian Pac J Cancer Prev.

[146] Salimi Z (2016). rs11895168 C allele and the increased risk of breast cancer in Isfahan population. Breast.

[147] Mansouri Bidkani M (2018). ErbB4 receptor polymorphism 2368A > C and risk of breast cancer. Breast.

[148] Bagheri F (2016). Tumor-promoting function of single nucleotide polymorphism rs1836724 (C3388T) alters multiple potential legitimate microRNA binding sites at the 3′-untranslated region of ErbB4 in breast cancer. Mol Med Rep.

[149] Normanno N (2006). Epidermal growth factor receptor (EGFR) signaling in cancer. Gene.

[150] Zhang XM (2003). Expression of tumor related genes NGX6, NAG-7, BRD7 in gastric and colorectal cancer. World J Gastroenterol.

[151] Wang L (2005). NGX6 gene inhibits cell proliferation and plays a negative role in EGFR pathway in nasopharyngeal carcinoma cells. J Cell Biochem.

[152] Eskandari-Nasab E (2012). Evaluation of UDP-glucuronosyltransferase 2B17 (UGT2B17) and dihydrofolate reductase (DHFR) genes deletion and the expression level of NGX6 mRNA in breast cancer. Mol Biol Rep.

[153] Naik A (2017). Neuropilin-1 associated molecules in the blood distinguish poor prognosis breast cancer: a cross-sectional study. Sci Rep.

[154] Seifi-Alan M (2018). Neuropilin-1 expression is associated with lymph node metastasis in breast cancer tissues. Cancer Manag Res.

[155] Cleveland RJ (2006). IGF1 CA repeat polymorphisms, lifestyle factors and breast cancer risk in the Long Island Breast Cancer Study Project. Carcinogenesis.

[156] Javadi M, Hematti S, Tavassoli M (2012). Polymorphic CA repeat length in insulin-like growth factor 1 and risk of breast cancer in Iranian women. Med Oncol.

[157] Vanhaesebroeck B, Waterfield MD (1999). Signaling by distinct classes of phosphoinositide 3-kinases. Exp Cell Res.

[158] Azizi Tabesh G (2017). The high frequency of PIK3CA mutations in iranian breast cancer patients. Cancer Invest.

[159] Sanaei S (2017). KRAS gene polymorphisms and their impact on breast cancer risk in an Iranian population. Asian Pac J Cancer Prev.

[160] Nazouri AS (2017). High expression of sphingosine kinase 1 in estrogen and progesterone receptors-negative breast cancer. Iran J Pathol.

[161] Bunney TD, Katan M (2010). Phosphoinositide signalling in cancer: beyond PI3K and PTEN. Nat Rev Cancer.

[162] Zhao L, Vogt PK (2008). Class I PI3K in oncogenic cellular transformation. Oncogene.

[163] Yuan TL, Cantley LC (2008). PI3K pathway alterations in cancer: variations on a theme. Oncogene.

[164] Heshmatpour N (2014). Association between the lengths of GT dinucleotide repeat in the PIK3CA gene with breast cancer risk. Med Oncol.

[165] Ghalaei A (2018). Overexpressed in colorectal carcinoma gene (OCC-1) upregulation and APPL2 gene downregulation in breast cancer specimens. Mol Biol Rep.

[166] Karami-Tehrani F (2016). Evaluation of RIP1K and RIP3K expressions in the malignant and benign breast tumors. Tumour Biol.

[167] Lipkowitz S (2003). The role of the ubiquitination-proteasome pathway in breast cancer: ubiquitin mediated degradation of growth factor receptors in the pathogenesis and treatment of cancer. Breast Cancer Res.

[168] Nikseresht M (2010). Overexpression of the novel human gene, UBE2Q2, in breast cancer. Cancer Genet Cytogenet.

[169] Vogl FD (2004). Glutathione S-transferases M1, T1, and P1 and breast cancer: a pooled analysis. Cancer Epidemiol Biomarkers Prev.

[170] Hashemi M (2012). Association between polymorphisms of glutathione S-transferase genes (GSTM1, GSTP1 and GSTT1) and breast cancer risk in a sample Iranian population. Biomark Med.

[171] Sharif MR (2017). Association of GSTO1 A140D and GSTO2 N142D gene variations with breast cancer risk. Asian Pac J Cancer Prev.

[172] Petersen DD (1991). Human CYP1A1 gene: cosegregation of the enzyme inducibility phenotype and an RFLP. Am J Hum Genet.

[173] Saadatian H (2014). Polymorphism of the cytochrome P-450 1A1 (A2455G) in women with breast cancer in Eastern Azerbaijan, Iran. Iran J Basic Med Sci.

[174] Saghafi F (2018). CYP2D6*3 (A2549del), *4 (G1846A), *10 (C100T) and *17 (C1023T) genetic polymorphisms in Iranian breast cancer patients treated with adjuvant tamoxifen. Biomed Rep.

[175] Darakhshan S (2013). Synergistic effects of tamoxifen and tranilast on VEGF and MMP-9 regulation in cultured human breast cancer cells. Asian Pac J Cancer Prev.

[176] Motamedi S (2012). Tamoxifen resistance and CYP2D6 copy numbers in breast cancer patients. Asian Pac J Cancer Prev.

[177] Meiyanto E, Hermawan A, Anindyajati A (2012). Natural products for cancer-targeted therapy: citrus flavonoids as potent chemopreventive agents. Asian Pac J Cancer Prev.

[178] Lash TL (2011). CYP2D6 inhibition and breast cancer recurrence in a population-based study in Denmark. J Natl Cancer Inst.

[179] Yazdi MF (2015). CYP2D6 genotype and risk of recurrence in tamoxifen treated breast cancer patients. Asian Pac J Cancer Prev.

[180] Ciocca DR (1993). Biological and clinical implications of heat shock protein 27,000 (Hsp27): a review. J Natl Cancer Inst.

[181] Mehlen P (1995). Constitutive expression of human hsp27, Drosophila hsp27, or human alpha B-crystallin confers resistance to TNF- and oxidative stress-induced cytotoxicity in stably transfected murine L929 fibroblasts. J Immunol.

[182] Calderwood SK (2006). Heat shock proteins in cancer: chaperones of tumorigenesis. Trends Biochem Sci.

[183] Vidyasagar A, Wilson NA, Djamali A (2012). Heat shock protein 27 (HSP27): biomarker of disease and therapeutic target. Fibrogenesis Tissue Repair.

[184] Homaei-Shandiz F (2016). Anti-heat shock protein-27 antibody levels in women with breast cancer: association with disease complications and two-year disease-free survival. Asian Pac J Cancer Prev.

[185] Bruhn O, Cascorbi I (2014). Polymorphisms of the drug transporters ABCB1, ABCG2, ABCC2 and ABCC3 and their impact on drug bioavailability and clinical relevance. Expert Opin Drug Metab Toxicol.

[186] Durmus S, Hendrikx JJ, Schinkel AH (2015). Apical ABC transporters and cancer chemotherapeutic drug disposition. Adv Cancer Res.

[187] Ghafouri H (2016). Association of ABCB1 and ABCG2 single nucleotide polymorphisms with clinical findings and response to chemotherapy treatments in Kurdish patients with breast cancer. Tumour Biol.

[188] Beauchemin N, Arabzadeh A (2013). Carcinoembryonic antigen-related cell adhesion molecules (CEACAMs) in cancer progression and metastasis. Cancer Metastasis Rev.

[189] Estiar MA (2017). High expression of CEACAM19, a new member of carcinoembryonic antigen gene family, in patients with breast cancer. Clin Exp Med.

[190] Thriveni K, Krishnamoorthy L, Ramaswamy G (2007). Correlation study of Carcino Embryonic Antigen & Cancer Antigen 15.3 in pretreated female breast cancer patients. Indian J Clin Biochem.

[191] Duffy MJ (1999). CA 15-3 and related mucins as circulating markers in breast cancer. Ann Clin Biochem.

[192] Moazzezy N (2014). Relationship between preoperative serum CA 15-3 and CEA levels and clinicopathological parameters in breast cancer. Asian Pac J Cancer Prev.

[193] Mansouri N (2016). Overexpression of the MUC1 gene in Iranian women with breast cancer micrometastasis. Asian Pac J Cancer Prev.

[194] Gumbiner BM (2005). Regulation of cadherin-mediated adhesion in morphogenesis. Nat Rev Mol Cell Biol.

[195] Tepass U (2000). Cadherins in embryonic and neural morphogenesis. Nat Rev Mol Cell Biol.

[196] Zarei F (2017). Higher risk of progressing breast cancer in Kurdish population associated to CDH1 -160 C/A polymorphism. EXCLI J.

[197] Naor D, Sionov RV, Ish-Shalom D (1997). CD44: structure, function, and association with the malignant process. Adv Cancer Res.

[198] Esmaeili R (2018). Unique CD44 intronic SNP is associated with tumor grade in breast cancer: a case control study and in silico analysis. Cancer Cell Int.

[199] Maetzel D (2009). Nuclear signalling by tumour-associated antigen EpCAM. Nat Cell Biol.

[200] Gottlinger HG (1986). The epithelial cell surface antigen 17-1A, a target for antibody-mediated tumor therapy: its biochemical nature, tissue distribution and recognition by different monoclonal antibodies. Int J Cancer.

[201] Meyer N, Penn LZ (2008). Reflecting on 25 years with MYC. Nat Rev Cancer.

[202] Sadeghi S, Hojati Z, Tabatabaeian H (2017). Cooverexpression of EpCAM and c-myc genes in malignant breast tumours. J Genet.

[203] Maguer-Satta V, Besancon R, Bachelard-Cascales E (2011). Concise review: neutral endopeptidase (CD10): a multifaceted environment actor in stem cells, physiological mechanisms, and cancer. Stem Cells.

[204] Taghizadeh-Kermani A (2014). The stromal overexpression of CD10 in invasive breast cancer and its association with clincophathologic factors. Iran J Cancer Prev.

[205] Fridolfsson HN (2014). Regulation of intracellular signaling and function by caveolin. FASEB J.

[206] Williams TM (2004). Caveolin-1 gene disruption promotes mammary tumorigenesis and dramatically enhances lung metastasis in vivo. Role of Cav-1 in cell invasiveness and matrix metalloproteinase (MMP-2/9) secretion. J Biol Chem.

[207] Fard ZT, Nafisi N (2018). The relationship between 6 polymorphisms of Caveolin-1 gene and the risk of breast cancer. Clin Breast Cancer.

[208] Mobasheri MB, Shirkoohi R, Modarressi MH (2016). Synaptonemal complex protein 3 transcript analysis in breast cancer. Iran J Public Health.

[209] Malek-Hosseini Z (2017). Elevated Syndecan-1 levels in the sera of patients with breast cancer correlate with tumor size. Breast Cancer.

[210] Rehman G (2014). Role of AMP-activated protein kinase in cancer therapy. Arch Pharm (Weinheim).

[211] Beavo JA (1995). Cyclic nucleotide phosphodiesterases: functional implications of multiple isoforms. Physiol Rev.

[212] Lincoln TM, Cornwell TL (1993). Intracellular cyclic GMP receptor proteins. FASEB J.

[213] Karami-Tehrani F (2012). Evaluation of PDE5 and PDE9 expression in benign and malignant breast tumors. Arch Med Res.

[214] Fiscus RR, Murad F (1988). cGMP-dependent protein kinase activation in intact tissues. Methods Enzymol.

[215] Karami-Tehrani F, Fallahian F, Atri M (2012). Expression of cGMP-dependent protein kinase, PKGIalpha, PKGIbeta, and PKGII in malignant and benign breast tumors. Tumour Biol.

[216] Moghbeli M (2016). Correlation of Wnt and NOTCH pathways in esophageal squamous cell carcinoma. J Cell Commun Signal.

[217] Moghbeli M (2015). Role of Msi1 and MAML1 in regulation of notch signaling pathway in patients with esophageal squamous cell carcinoma. J Gastrointest Cancer.

[218] Paryan M (2016). Over-expression of NOTCH1 as a biomarker for invasive breast ductal carcinoma. 3 Biotech.

[219] Moghbeli M (2016). Role of Msi1 and PYGO2 in esophageal squamous cell carcinoma depth of invasion. J Cell Commun Signal.

[220] Taghavi A (2016). Gene expression profiling of the 8q22-24 position in human breast cancer: TSPYL5, MTDH, ATAD2 and CCNE2 genes are implicated in oncogenesis, while WISP1 and EXT1 genes may predict a risk of metastasis. Oncol Lett.

[221] Moghbeli M (2014). Cancer stem cell detection and isolation. Med Oncol.

[222] Balicki D (2007). Moving forward in human mammary stem cell biology and breast cancer prognostication using ALDH1. Cell Stem Cell.

[223] Ginestier C (2007). ALDH1 is a marker of normal and malignant human mammary stem cells and a predictor of poor clinical outcome. Cell Stem Cell.

[224] Madjd Z (2012). High expression of stem cell marker ALDH1 is associated with reduced BRCA1 in invasive breast carcinomas. Asian Pac J Cancer Prev.

[225] Mandlekar S, Kong AN (2001). Mechanisms of tamoxifen-induced apoptosis. Apoptosis.

[226] Yu FL, Bender W (2002). A proposed mechanism of tamoxifen in breast cancer prevention. Cancer Detect Prev.

[227] Musgrove EA, Sutherland RL (2009). Biological determinants of endocrine resistance in breast cancer. Nat Rev Cancer.

[228] Eccles MR (2002). PAX genes in development and disease: the role of PAX2 in urogenital tract development. Int J Dev Biol.

[229] Jahangiri R (2018). PAX2 expression is correlated with better survival in tamoxifen-treated breast carcinoma patients. Tissue Cell.

[230] Vervoorts J, Luscher-Firzlaff J, Luscher B (2006). The ins and outs of MYC regulation by posttranslational mechanisms. J Biol Chem.

[231] Qu X (2002). Characterization and expression of three novel differentiation-related genes belong to the human NDRG gene family. Mol Cell Biochem.

[232] Zhao W (2001). Cloning and expression pattern of the human NDRG3 gene. Biochim Biophys Acta.

[233] Estiar MA (2017). Clinical significance of NDRG3 in patients with breast cancer. Future Oncol.

[234] Hosseini A (2014). *Estrogen receptor alpha gene expression in breast cancer tissues from the Iranian population*–*a pilot study*. Asian Pac J Cancer Prev.

[235] Abbasi S (2013). Association of estrogen receptor-alpha A908G (K303R) mutation with breast cancer risk. Int J Clin Exp Med.

[236] Farzaneh F (2016). Analysis of CYP17, CYP19 and CYP1A1 Gene polymorphisms in Iranian women with breast cancer. Asian Pac J Cancer Prev.

[237] Duncan LJ, Coldham NG, Reed MJ (1994). The interaction of cytokines in regulating oestradiol 17 beta-hydroxysteroid dehydrogenase activity in MCF-7 cells. J Steroid Biochem Mol Biol.

[238] Newman SP (2000). Regulation of steroid sulphatase expression and activity in breast cancer. J Steroid Biochem Mol Biol.

[239] Danforth DN, Sgagias MK (1993). Tumour necrosis factor-alpha modulates oestradiol responsiveness of MCF-7 breast cancer cells in vitro. J Endocrinol.

[240] Roodi N (1995). Estrogen receptor gene analysis in estrogen receptor-positive and receptor-negative primary breast cancer. J Natl Cancer Inst.

[241] Kamali-Sarvestani E (2005). Association of TNF-alpha and TNF-beta gene polymorphism with steroid receptor expression in breast cancer patients. Pathol Oncol Res.

[242] Di C (2015). Basophil-associated OX40 ligand participates in the initiation of Th2 responses during airway inflammation. J Biol Chem.

[243] Lee J (2008). Activated B cells modified by electroporation of multiple mRNAs encoding immune stimulatory molecules are comparable to mature dendritic cells in inducing in vitro antigen-specific T-cell responses. Immunology.

[244] Ohshima Y (1997). Expression and function of OX40 ligand on human dendritic cells. J Immunol.

[245] Vakil R, Mashayekhi F (2018). OX40L gene polymorphism and breast cancer in Iranian population. Exp Oncol.

[246] Crew KD (2013). Vitamin d: are we ready to supplement for breast cancer prevention and treatment?. ISRN Oncol.

[247] Jurutka PW (2001). Molecular nature of the vitamin D receptor and its role in regulation of gene expression. Rev Endocr Metab Disord.

[248] Thorne J, Campbell MJ (2008). The vitamin D receptor in cancer. Proc Nutr Soc.

[249] Barsony J, Prufer K (2002). Vitamin D receptor and retinoid X receptor interactions in motion. Vitam Horm.

[250] Colagar AH, Firouzjah HM, Halalkhor S (2015). Vitamin D Receptor Poly(A) Microsatellite Polymorphism and 25-Hydroxyvitamin D Serum Levels: association with Susceptibility to Breast Cancer. J Breast Cancer.

[251] Anderson MG (2006). Expression of VDR and CYP24A1 mRNA in human tumors. Cancer Chemother Pharmacol.

[252] Anderson PH (2003). Quantification of mRNA for the vitamin D metabolizing enzymes CYP27B1 and CYP24 and vitamin D receptor in kidney using real-time reverse transcriptase- polymerase chain reaction. J Mol Endocrinol.

[253] Haussler MR (2013). Molecular mechanisms of vitamin D action. Calcif Tissue Int.

[254] Horvath HC (2010). The candidate oncogene CYP24A1: a potential biomarker for colorectal tumorigenesis. J Histochem Cytochem.

[255] Zhalehjoo N, Shakiba Y, Panjehpour M (2017). Gene expression profiles of CYP24A1 and CYP27B1 in malignant and normal breast tissues. Mol Med Rep.

[256] Shahabi A (2018). Vitamin D receptor gene polymorphism: association with susceptibility to early-onset breast cancer in Iranian, BRCA1/2-mutation carrier and non-carrier patients. Pathol Oncol Res.

[257] Shahbazi S (2013). BsmI but not FokI polymorphism of VDR gene is contributed in breast cancer. Med Oncol.

[258] Reid AH (2008). CYP17 inhibition as a hormonal strategy for prostate cancer. Nat Clin Pract Urol.

[259] Setiawan VW (2007). CYP17 genetic variation and risk of breast and prostate cancer from the National Cancer Institute Breast and Prostate Cancer Cohort Consortium (BPC3). Cancer Epidemiol Biomarkers Prev.

[260] Ebrahimi E (2017). CYP17 MspA1 gene polymorphism and breast cancer patients according to age of onset in cancer institute of Iran. Iran J Public Health.

[261] Scanlan MJ, Simpson AJ, Old LJ (2004). The cancer/testis genes: review, standardization, and commentary. Cancer Immun.

[262] Salmaninejad A (2016). Cancer/testis antigens: expression, regulation, tumor invasion, and use in immunotherapy of cancers. Immunol Invest.

[263] Esmaeili R (2015). AKAP3 correlates with triple negative status and disease free survival in breast cancer. BMC Cancer.

[264] Lim SK (2011). Tyrosine phosphorylation of transcriptional coactivator WW-domain binding protein 2 regulates estrogen receptor alpha function in breast cancer via the Wnt pathway. FASEB J.

[265] Nourashrafeddin S (2014). Expression analysis of PAWP during mouse embryonic stem cell-based spermatogenesis in vitro. Vitro Cell Dev Biol Anim.

[266] Nourashrafeddin S (2015). Elevated expression of the testis-specific gene WBP2NL in breast cancer. Biomark Cancer.

[267] Rastgoosalami M (2016). Evaluation of MAGE-1 cancer-testis antigen expression in invasive breast cancer and its correlation with prognostic factors. Iran J Cancer Prev.

[268] Dianatpour M (2012). Expression of testis specific genes TSGA10, TEX101 and ODF3 in breast cancer. Iran Red Crescent Med J.

[269] Nakagawa Y (2001). Outer dense fiber 2 is a widespread centrosome scaffold component preferentially associated with mother centrioles: its identification from isolated centrosomes. Mol Biol Cell.

[270] Shibata-Minoshima F (2012). Identification of RHOXF2 (PEPP2) as a cancer-promoting gene by expression cloning. Int J Oncol.

[271] Zou Y, Zhong W (2012). A likely role for a novel PH-domain containing protein, PEPP2, in connecting membrane and cytoskeleton. Biocell.

[272] Kazemi-Oula G (2015). Upregulation of RHOXF2 and ODF4 expression in breast cancer tissues. Cell J.

[273] Hsu CC, Tseng LM, Lee HC (2016). Role of mitochondrial dysfunction in cancer progression. Exp Biol Med (Maywood).

[274] Zong WX, Rabinowitz JD, White E (2016). Mitochondria and Cancer. Mol Cell.

[275] Yadav N, Chandra D (2013). Mitochondrial DNA mutations and breast tumorigenesis. Biochim Biophys Acta.

[276] Damm F (2012). Prognostic implications and molecular associations of NADH dehydrogenase subunit 4 (ND4) mutations in acute myeloid leukemia. Leukemia.

[277] Gogvadze V, Orrenius S, Zhivotovsky B (2008). Mitochondria in cancer cells: what is so special about them?. Trends Cell Biol.

[278] Arezi P, Rezvani Z (2018). The variation of mitochondrial NADH dehydrogenase subunit 4 (mtND4) and molecular dynamics simulation of SNPs among Iranian women with breast cancer. J Mol Graph Model.

[279] Czarnecka AM (2010). Mitochondrial DNA mutations in cancer–from bench to bedside. Front Biosci (Landmark Ed).

[280] Park JS (2009). A heteroplasmic, not homoplasmic, mitochondrial DNA mutation promotes tumorigenesis via alteration in reactive oxygen species generation and apoptosis. Hum Mol Genet.

[281] Jonckheere AI, Smeitink JA, Rodenburg RJ (2012). Mitochondrial ATP synthase: architecture, function and pathology. J Inherit Metab Dis.

[282] Ghaffarpour M (2014). The mitochondrial ATPase6 gene is more susceptible to mutation than the ATPase8 gene in breast cancer patients. Cancer Cell Int.

